# Multiscale imaging of therapeutic anti-PD-L1 antibody localization using molecularly defined imaging agents

**DOI:** 10.1186/s12951-022-01272-5

**Published:** 2022-02-02

**Authors:** Iris M. Hagemans, Peter J. Wierstra, Kas Steuten, Janneke D. M. Molkenboer-Kuenen, Duco van Dalen, Martin ter Beest, Johan M. S. van der Schoot, Olga Ilina, Martin Gotthardt, Carl G. Figdor, Ferenc A. Scheeren, Sandra Heskamp, Martijn Verdoes

**Affiliations:** 1 Department of Tumor Immunology, Radboud Institute for Molecular Life Sciences, Radboud University Medical Center, Nijmegen, The Netherlands; 2 Institute for Chemical Immunology, Nijmegen, The Netherlands; 3 Department of Medical Imaging, Nuclear Medicine, Radboud Institute for Molecular Life Sciences, Radboud University Medical Center, Nijmegen, The Netherlands; 4 Division of Immunotherapy, Oncode Institute, Radboud University Medical Center, Nijmegen, The Netherlands; 5 Department of Dermatology, Leiden University Medical Centre, Leiden, The Netherlands

**Keywords:** Fluorescence imaging, Nuclear imaging, Immune checkpoints, Antibody, Cancer

## Abstract

**Background:**

While immune checkpoint inhibitors such as anti-PD-L1 antibodies have revolutionized cancer treatment, only subgroups of patients show durable responses. Insight in the relation between clinical response, PD-L1 expression and intratumoral localization of PD-L1 therapeutics could improve patient stratification. Therefore, we present the modular synthesis of multimodal antibody-based imaging tools for multiscale imaging of PD-L1 to study intratumoral distribution of PD-L1 therapeutics.

**Results:**

To introduce imaging modalities, a peptide containing a near-infrared dye (sulfo-Cy5), a chelator (DTPA), an azide, and a sortase-recognition motif was synthesized. This peptide and a non-fluorescent intermediate were used for site-specific functionalization of c-terminally sortaggable mouse IgG1 (mIgG1) and Fab anti-PD-L1. To increase the half-life of the Fab fragment, a 20 kDa PEG chain was attached via strain-promoted azide-alkyne cycloaddition (SPAAC). Biodistribution and imaging studies were performed with ^111^In-labeled constructs in 4T1 tumor-bearing mice. Comparing our site-specific antibody-conjugates with randomly conjugated antibodies, we found that antibody clone, isotype and method of DTPA conjugation did not change tumor uptake. Furthermore, addition of sulfo-Cy5 did not affect the biodistribution. PEGylated Fab fragment displayed a significantly longer half-life compared to unPEGylated Fab and demonstrated the highest overall tumor uptake of all constructs. PD-L1 in tumors was clearly visualized by SPECT/CT, as well as whole body fluorescence imaging. Immunohistochemistry staining of tumor sections demonstrated that PD-L1 co-localized with the fluorescent and autoradiographic signal. Intratumoral localization of the imaging agent could be determined with cellular resolution using fluorescent microscopy.

**Conclusions:**

A set of molecularly defined multimodal antibody-based PD-L1 imaging agents were synthesized and validated for multiscale monitoring of PD-L1 expression and localization. Our modular approach for site-specific functionalization could easily be adapted to other targets.

**Graphical Abstract:**

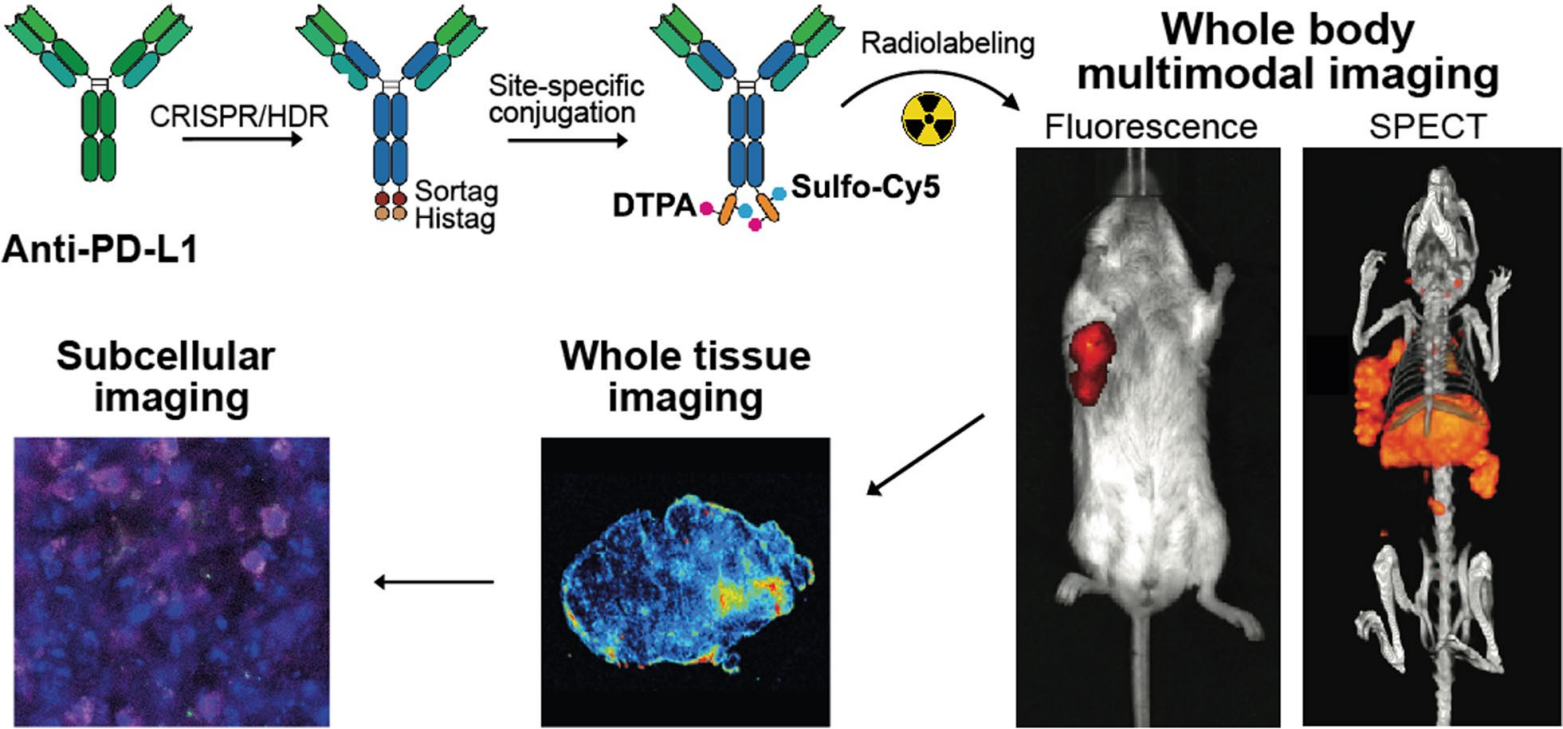

**Supplementary Information:**

The online version contains supplementary material available at 10.1186/s12951-022-01272-5.

## Background

Over the past decade, immunotherapy, in particular blockade of immune checkpoint molecules such as Programmed Death 1 (PD-1) and its ligand PD-L1 using antibodies, has revolutionized the field of cancer therapeutics due to unparalleled responses in a range of cancers [1–3]. Despite these successes, a large number of patients does not benefit from this treatment [4]. Moreover, anti-PD-L1 (aPD-L1) treatment is associated with significant immune-related side effects [5–8] and comes with a high economic burden on society [9]. PD-L1 immunohistochemistry (IHC) has been shown to be a partial predictor for response [10]. However, not all patients selected using this biomarker actually respond to treatment. Therefore, there is a clear need to better understand the dynamics of response to PD-L1 blockade. Currently, IHC on tumor biopsies is the gold standard for characterization of the tumor microenvironment even though it has significant downsides. First of all, IHC of biopsy material yields limited spatial information as PD-L1 expression can be heterogeneous within and between tumor lesions and does not provide information about the accessibility for PD-L1 therapeutics [11]. In contrast, non-invasive whole-body nuclear imaging of PD-L1 allows for assessment of multiple lesions concurrently and if desired over time also taking into account target accessibility. However, the resulting images have a limited spatial resolution, which does not allow for tissue analysis on a cellular level to differentiate between cell populations that express the targeted molecule [12]. This is relevant because in some tumors therapeutic outcome has been linked to intratumoral PD-L1 expression by tumor-infiltrating immune cells, whereas PD-L1 was not expressed by tumor cells [13, 14]. However, others have reported the reverse, indicating a context dependency for this correlation [15, 16]. Therefore, whole-body imaging would ideally be supplemented with an imaging modality suitable to assess the distribution of PD-L1 therapeutics at a cellular level, such as a fluorescent dye. This allows microscopic imaging at high spatial resolution [17].

Several preclinical [18–21] and clinical [22, 23] imaging studies have been performed using different PD-L1-targeting agents. In most of these studies, imaging moieties were coupled to the targeting antibody using non-selective protein modification methods, without spatial control. However, such random conjugation can interfere with the antigen-binding region and consequently alter the binding affinity [24] and in vivo pharmacokinetics [25, 26]. In contrast, site-specific labeling does not interfere with the antigen-binding site and yields a more controlled, homogeneous and therefore reproducible product. Recently, we developed an approach to modify antibodies site-specifically by applying the CRISPR/HDR technology to PD-L1 hybridoma cells [27]. We generated Fab fragments and chimeric antibodies with switched mouse Fc isotypes, while at the same time introducing a sortag [28, 29] at the C-terminus of the heavy chain. This tag enables site-specific chemo-enzymatic functionalization of these antibody products. Isotype switching is relevant in the context of imaging, since multiple doses of a foreign antibody can elicit an immune response directed against the Fc region [30–33], impacting longitudinal monitoring of a target. Moreover, small antibody formats like Fab fragments lack the Fc region entirely and can be of interest due to their relatively short half-life [34–38] and potentially increased ability to penetrate tumor tissue [39–41]. Unfortunately, a fast clearance rate can also lead to low tumor retention [35], which can be attenuated by conjugation to a polyethyleneglycol (PEG) chain [42–45].

**Fig. 1:**
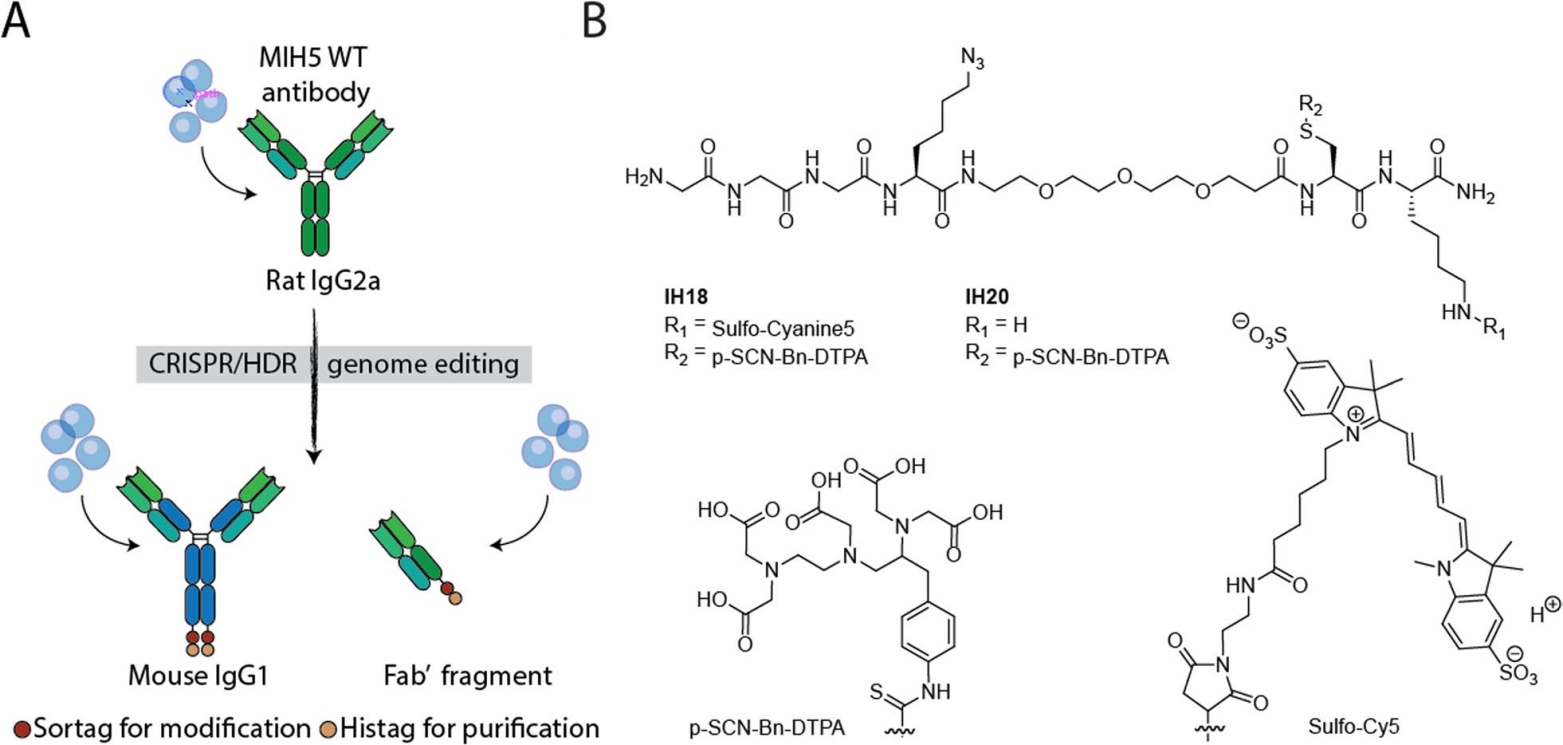
Molecular toolbox of site-specific functionalizable anti-PD-L1 antibody formats and imaging peptides.** A** Application of the CRISPR/HDR strategy to anti-PD-L1 hybridoma MIH5 created a sortaggable Fab fragment and chimeric mouse IgG1 monoclonal antibody against PD-L1. **B** Molecular structure of imaging peptides IH20 and IH18

Here, we demonstrate that combining our CRISPR/HDR hybridoma engineering technology with chemical synthesis provides a plug-and-play platform to create molecularly defined antibody-based multimodal, multiscale PD-L1 imaging agents. To this end, we functionalized engineered mouse IgG1 (mIgG1) and Fab aPD-L1 site-specifically with a peptide containing both the radionuclide chelator diethylenetriaminepentaacetic acid anhydride (DTPA) and near-infrared (NIR) dye sulfo-Cy5 (Fig. 1). Additionally, an azide was included in the peptide to allow for further functionalization through strain-promoted azide-alkyne cycloaddition (SPAAC). This way, we modified our Fab fragment with a 20 kDa PEG chain to increase the half-life in vivo. We show that our agents allow for whole-body SPECT imaging as well as tissue-level fluorescence microscopy imaging. To the best of our knowledge, this is the first example of multimodal and multiscale imaging to assess the intratumoral distribution of PD-L1 therapeutics.

## Results

### The effect of isotype switching and site-specific conjugation on the in vivo behavior of aPD-L1 imaging agents

**Fig. 2:**
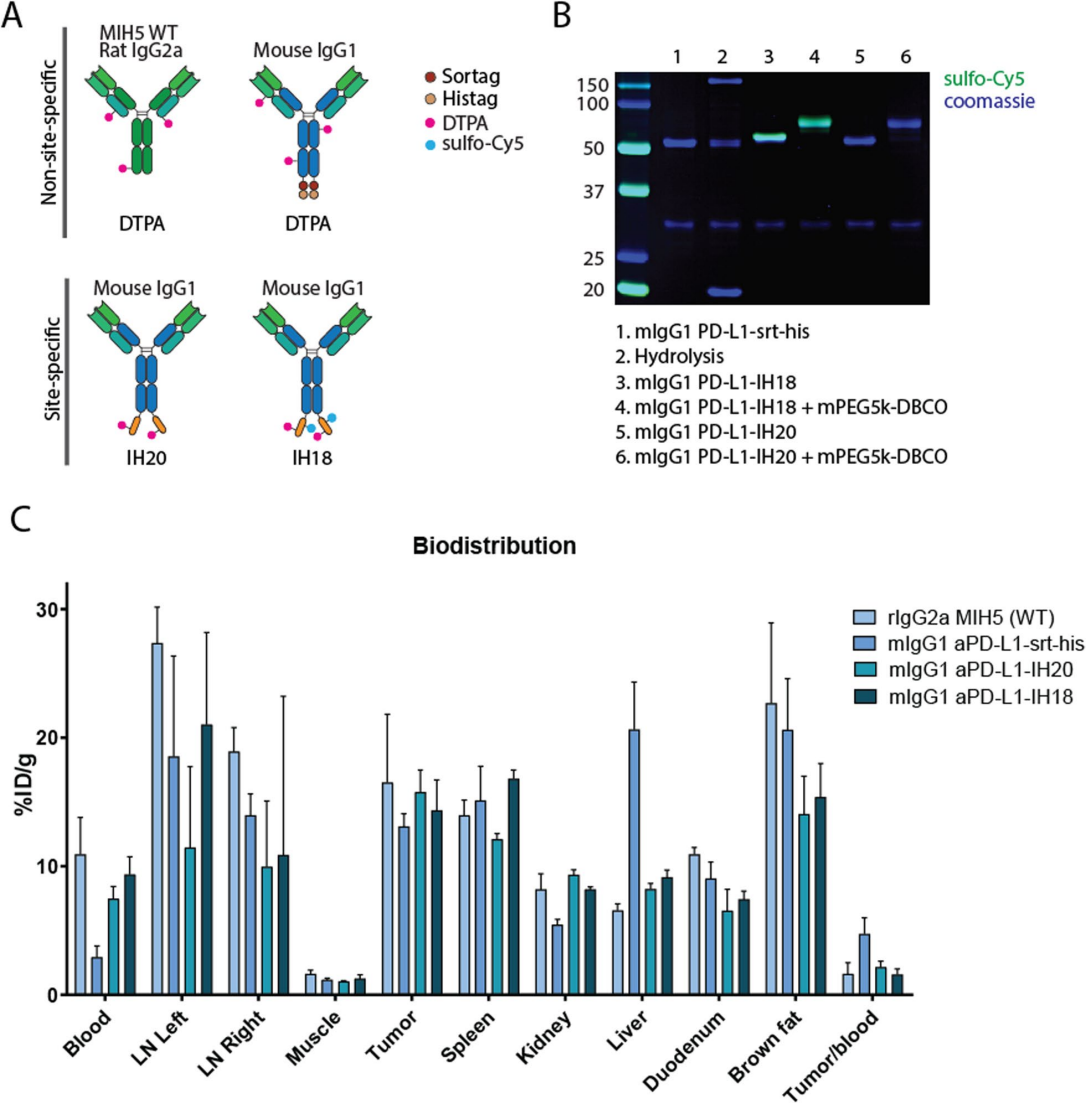
Biodistribution of anti-PD-L1 monoclonal antibody panel. **A** WT MIH5 antibody and mouse (mIgG1) anti-PD-L1 antibodies were labeled non-site-specifically with DTPA. mIgG1 was labeled site-specifically with IH20 and IH18, where IH20 contains DTPA alone and IH18 contains an additional sulfo-Cy5. **B** SDS-PAGE analysis of purified site-specifically labeled antibodies. An analytical fraction of mIgG1-IH18 and mIgG1-IH20 was reacted with 5 kDa mPEG-DBCO to analyze purity and azide functionality. This resulted in the near-quantitative conversion to a product of higher molecular weight. **(C)** Mice bearing orthotopic 4T1 tumors were injected with one of four different ^111^In-labeled anti-PD-L1 antibodies. Biodistribution was determined ex vivo 24 h after injection. Values are presented as percentage injected dose per gram (%ID/g) and shown as mean ± SD, n = 5

First, using our molecular toolbox (Fig. 1A and B) we set out to determine the consequence of isotype switching of the PD-L1 targeting antibody (clone MIH5) from rIgG2a to mIgG1 [27], as well as random versus site-specific radiolabeling and additional functionalization with a fluorescent dye, on in vivo biodistribution of these compounds (Fig. 2A). To facilitate the incorporation of imaging moieties in a site-specific way, we synthesized the DTPA equipped nuclear imaging peptide **IH20** and the multimodal nuclear and NIR imaging peptide **IH18**, both containing an N-terminal triglycine motif (Fig. 1B and Additional file 1: Fig. S1). After production of the ‘wild type’ rIgG2a (rIgG2a WT) and the c-terminal sortag-histag carrying mIgG1 chimeric aPD-L1 (aPD-L1 mIgG1-srt-his), we site-specifically conjugated peptides **IH20** and **IH18** to the latter antibody to obtain aPD-L1 **mIgG1-IH20** and **mIgG1-IH18** (70% isolated yield, Fig. 2b). For non-site-specific DTPA conjugation we exposed aPD-L1 mIgG1-srt-his and rIgG2a WT to an excess of S-2-(4-Isothiocyanatobenzyl)-DTPA (p-SCN-Bn-DTPA). Having the four conjugates in hand, we labeled them with ^111^In and determined the biodistribution in 4T1 tumor-bearing mice. At 24 h p.i., tumor uptake was similar for all constructs (± 15% ID/g, Fig. 2C, Additional file 1: Table S1). Radiolabeled isotype switched mIgG1 PD-L1-srt-his antibody showed a twofold increase in hepatic uptake compared with the WT rIgG2a antibody (20.65 ± 3.67% ID/g vs 6.56 ± 0.51% ID/g, *p* < 0.0001), while blood concentration and kidney uptake were significantly lower (2.91 ± 0.88% ID/g and 10.94 ± 2.86% ID/g for blood; 5.44 ± 0.43% ID/g and 8.19 ± 1.21% ID/g for kidneys, *p* < 0.0001). Site-specifically conjugated isotype-switched mIgG1 with either DTPA alone (**mIgG1-IH20**) or with the additional fluorophore (**mIgG1-IH18**) did not show a significant change in blood clearance or liver uptake, compared to randomly labeled WT rIgG2a antibody. Spleen uptake of **mIgG1-IH18** was increased compared to **mIgG1-IH20** (16.80 ± 0.68% ID/g vs 12.11 ± 0.43% ID/g, *p* = 0.0015). Duodenum uptake was slightly decreased for IH18- and IH20-labeled antibodies (7.42 ± 0.64% ID/g, *p* = 0.0009, 6.53 ± 1.69% ID/g, *p* < 0.0001) compared to the MIH5 WT antibody (10.94 ± 0.52% ID/g). Taken together, isotype switching and site-specific conjugation of PD-L1 targeting antibody with **IH18** and **IH20** was feasible without major changes in the tumor targeting.

### Site-specific multimodal PD-L1 imaging conjugates with different pharmacokinetics

**Fig. 3:**
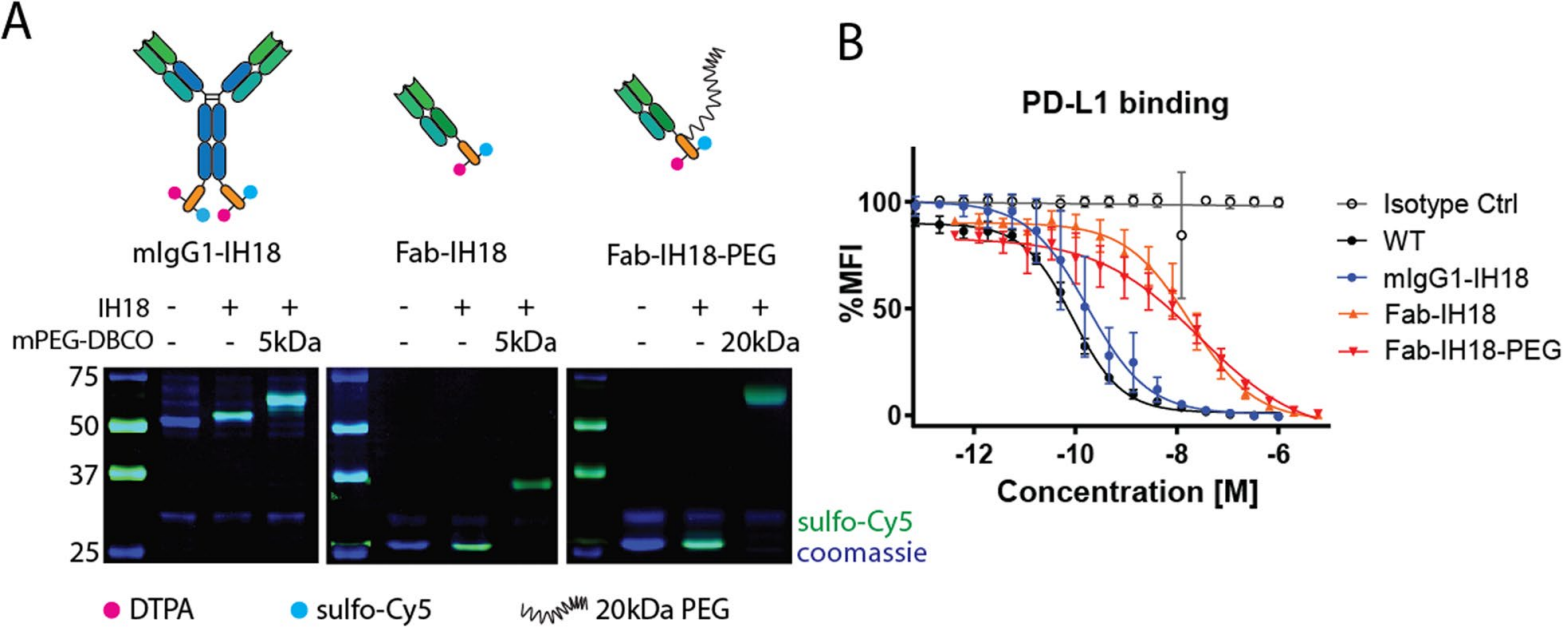
In vitro characterization of multimodal PD-L1 imaging tools. **A** SDS-PAGE analysis of the antibody formats before and after sortagging with IH18. After purification using NiNTA beads and SEC, an analytical fraction of mIgG1-IH18 and Fab-IH18 was reacted with 5 kDa mPEG-DBCO to analyze purity and azide functionality. This resulted in the near-quantitative conversion to a fluorescent product of higher molecular weight. Fab-IH18-PEG was produced through large-scale reaction of Fab-IH18 with 20 kDa mPEG-DBCO and subsequently purified using cation exchange chromatography. **B** Competition assay. Renca cells were incubated with a serial dilution of unlabeled anti-PD-L1 MIH5 (WT) or different antibody-conjugate (mIgG1-IH18, Fab-IH18, Fab-IH18-PEG) concentrations, as well as commercially available anti-PD-L1-PE. Data are presented as mean ± SD, n = 3

Having demonstrated the ability to produce site-specifically functionalized aPD-L1 mIgG1 chimera with **IH18**, we next aimed to use our platform to produce multimodal imaging conjugates with different pharmacokinetic properties. In addition to **mIgG1-IH18**, we produced c-terminal sortag-histag carrying aPD-L1 Fab fragments [27] to conjugate **IH18** in a sortase catalyzed reaction to obtain **Fab-IH18** (75% isolated yield, Fig. 3A and Additional file 1: Fig. S4). To prolong the anticipated short half-life, we reacted the azide functionality in **Fab-IH18** with 20 kDa mPEG-DBCO to obtain **Fab-IH18-PEG** (60% isolated yield, Fig. 3A and Additional file 1: Fig S3C).

#### In vitro characteristics

To validate preservation of antigen specificity, we carried out a competitive binding assay for **mIgG1-IH18**, **Fab-IH18** and **Fab-IH18-PEG**. All site-specifically conjugated constructs and the WT rIgG2a competed with a commercially available fluorescently labeled MIH5 aPD-L1 antibody for target binding in a concentration-dependent manner, indicating post-conjugation antigen specificity (Fig. 3B). Moreover, IC_50_ values were similar for mIgG1 PD-L1-IH18 (180 pM) and the unconjugated WT antibody (88 pM). The IC_50_ values of the Fab fragments were ± 100-fold higher compared to **mIgG1-IH18** (18.3 nM and 27.8 nM for **Fab-IH18** and **Fab-IH18-PEG**, respectively). Direct monitoring of binding to target cells measuring sulfo-Cy5 fluorescence reflected the results of the competitive binding assay (Fig. S5A). Finally, all ^111^In radiolabeled antibody-conjugates showed target-specific binding and internalization over time (Additional file 1: Fig. S5B-D). These data suggest a relatively high internalization rate for **mIgG1-IH18** and **Fab-IH18-PEG** compared to **Fab-IH18**.

#### Biodistribution in tumor-bearing mice

**Fig. 4:**
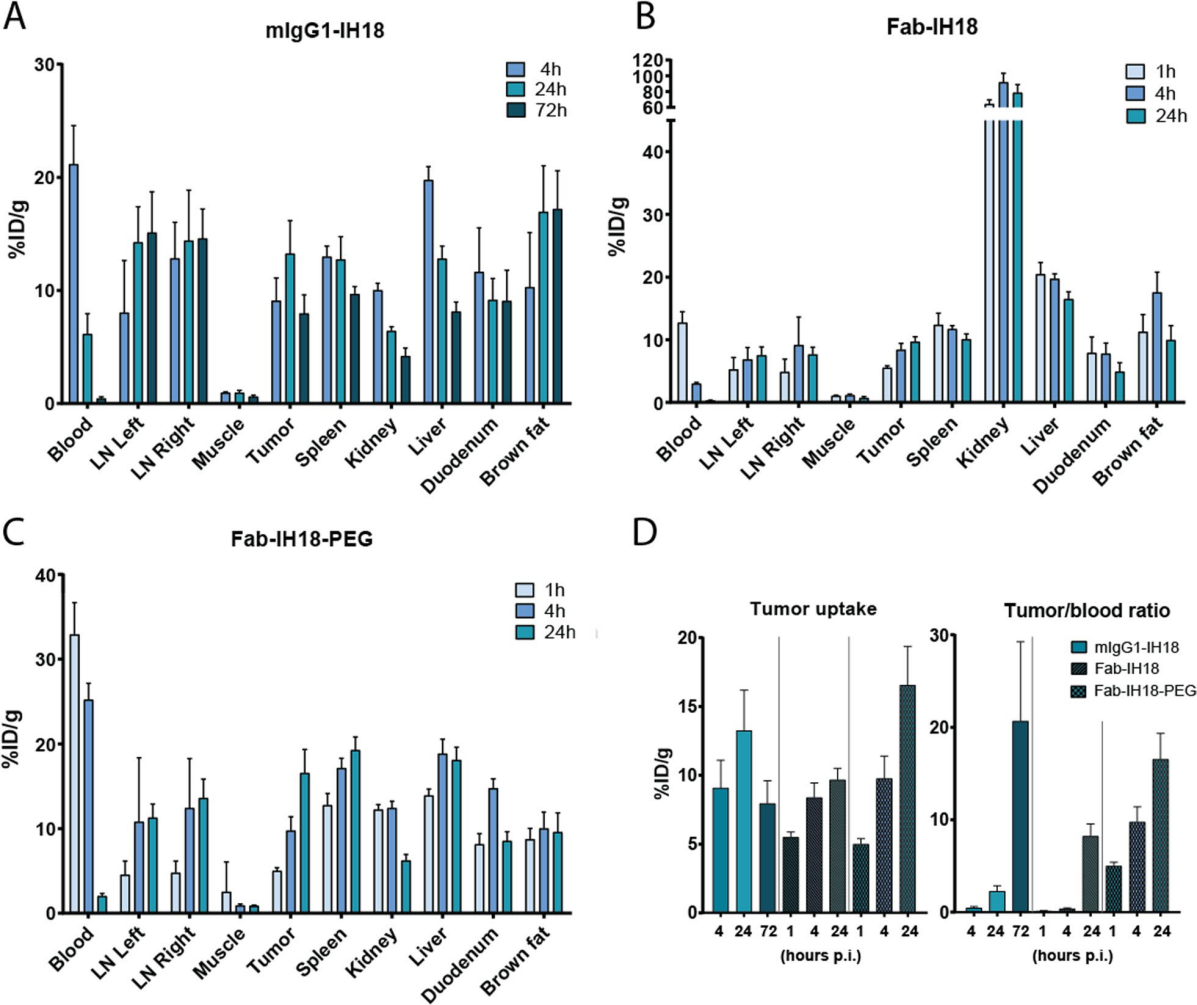
Biodistribution results of anti-PD-L1 multimodal imaging tools. Biodistribution is shown at several time points after injection (p.i.) of ^111^In-labeled **A** mIgG1-IH18, **B** Fab-IH18 or **C** Fab-IH18-PEG. **D** Tumor uptake values (left graph) and tumor/blood ratios (right graph) of the three constructs are compared directly at each time point. Data are shown as mean ± SD, n = 5

The biodistribution of the radiolabeled multimodal aPD-L1 site-specific conjugates was assessed in 4T1 tumor-bearing mice 4 h, 24 h and 72 h p.i. for **mIgG1-IH18** and 1 h, 4 h and 24 h p.i. for **Fab-IH18** and **Fab-IH18-PEG** (Fig. 4 and Additional file 1: Table S2). For **mIgG1-IH18**, tumor uptake was highest at 24 h p.i. (13.24 ± 2.93%ID/g). However, of the timepoints measured, the optimum tumor/blood ratio (TBR) was achieved 72 h p.i. (20.67 ± 8.60) due to high concentrations in blood at the 24 h time point (6.13 ± 1.81%ID/g). **Fab-IH18** was cleared from the circulation more rapidly, demonstrated by a low concentration in the blood 24 h p.i. (0.31 ± 0.08%ID/g). Highest TBR and tumor accumulation was observed at 24 h p.i. (32.68 ± 7.95; 9.67 ± 0.84%ID/g respectively). **Fab-IH18** displayed a high kidney accumulation (78.36 ± 10.31%ID/g), due to renal clearance of the low molecular weight conjugate. In contrast, **Fab-IH18-PEG** showed increased circulation time compared with **Fab-IH18** (the concentration 24 h p.i. was 2.03 ± 0.30%ID/g), causing the TBR to remain lower than for the other two constructs (8.22 ± 1.32, *p* = 0.0005 and *p* < 0.0001 compared to mIgG1 after 72 h and Fab after 24 h, respectively), while tumor accumulation for **Fab-IH18-PEG** after 24 h was significantly higher than **Fab-IH18** (16.55 ± 2.80%ID/g, p < 0.0001). Taken together the different conjugates demonstrated high tumor uptake with TBR in accordance with clearance kinetics of the antibody formats used.

#### Multiscale multimodal PD-L1 imaging

**Fig. 5:**
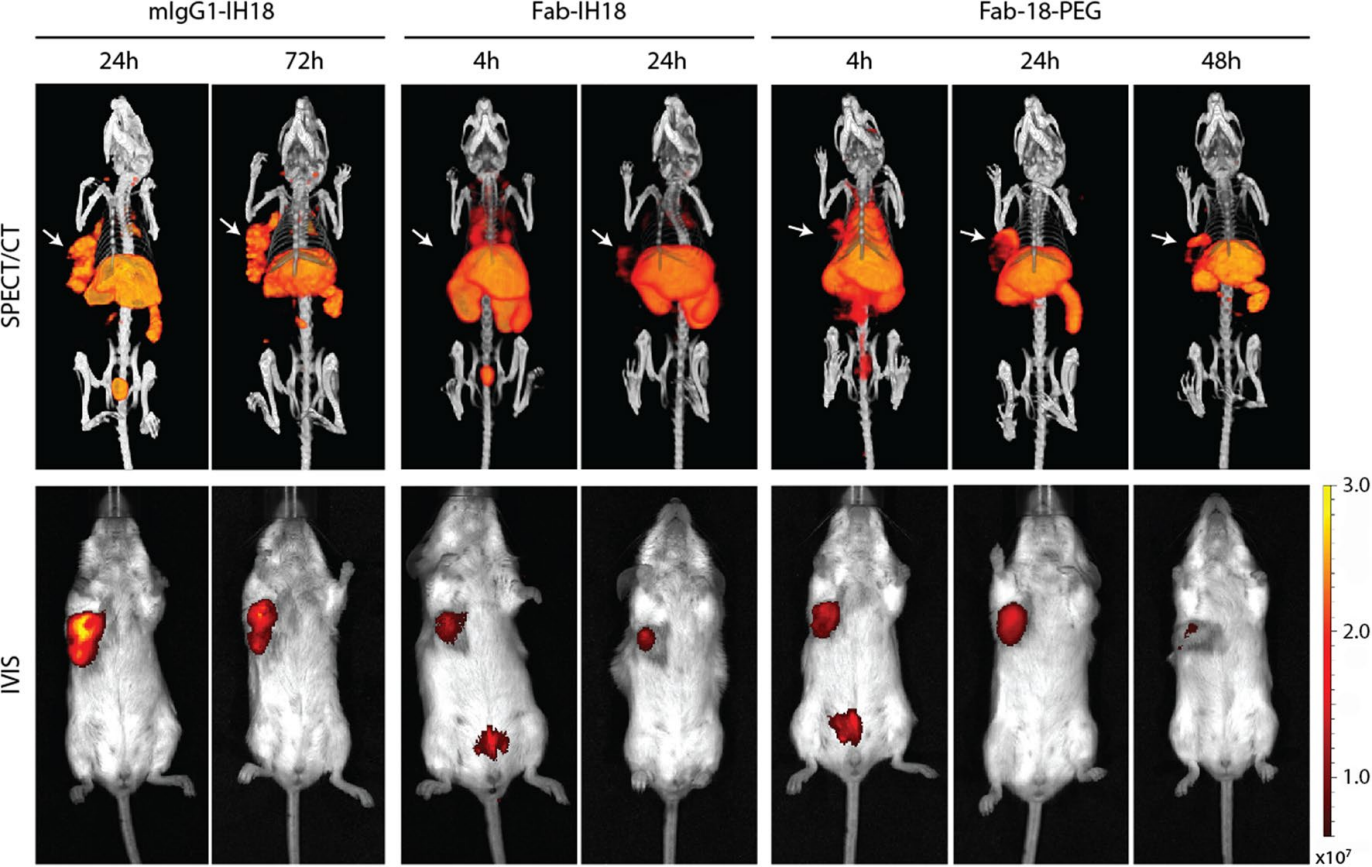
SPECT/CT and IVIS imaging of labeled antibody distribution in mice bearing orthotopic 4T1 tumors. Representative examples of SPECT/CT and IVIS scans of mice bearing 4T1 tumors, acquired at different time points after injection of ^111^In-labeled mIgG1-IH18, Fab-IH18 or Fab-IH18-PEG. Tumors are indicated with white arrows. Fluorescence intensity is indicated as radiant efficiency [(p/s/cm^2^/sr)/(μW/cm^2^)]

**Fig. 6:**
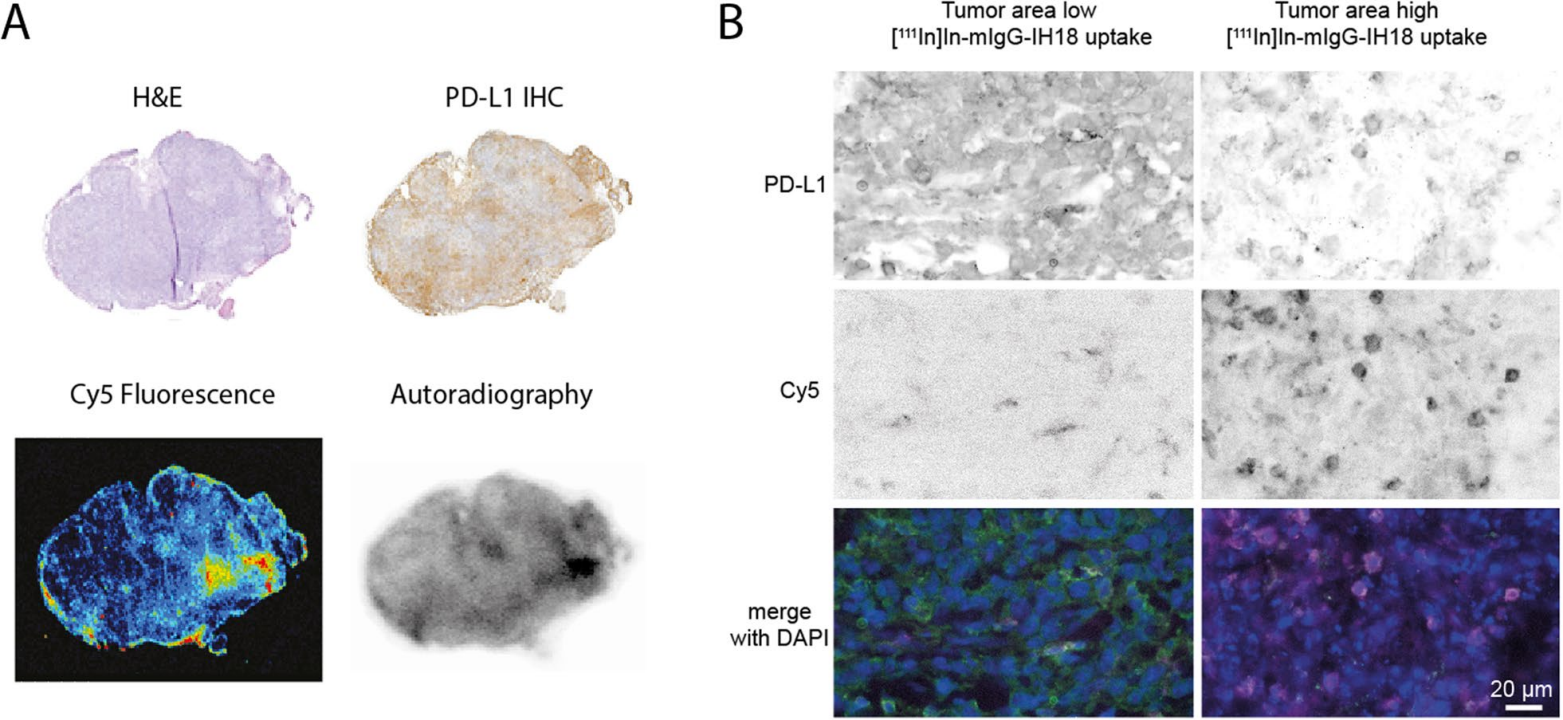
Analysis of tumor sections shows distribution of mIgG1-IH18 radiographic and fluorescent signal and PD-L1 expression.** A** Physically adjacent tissue sections of a tumor from a mIgG1-IH18 treated mouse are stained with H&E, PD-L1 immunostaining, or where fluorescent and radiographic signal is acquired. PD-L1, autoradiography and fluorescence patterns are overlapping, demonstrating co-localization of these signals. **B** Fluorescence microscopy shows localization of mIgG1-IH18 with cellular resolution (DAPI in blue, Cy5 fluorescence in purple and PD-L1 immunofluorescent staining in green in the merge image)

Next, we performed SPECT/CT and IVIS imaging in mice bearing orthotopic 4T1 tumors using our ^111^In-labeled PD-L1 multimodal imaging conjugates (Fig. 5, Additional file 1: Fig. S6). Based on the previous biodistribution results (Fig. 4), imaging was performed at 24 and 72 h for **mIgG1-IH18**, and at 4 and 24 h for **Fab-IH18.** For **Fab-IH18-PEG** we added a 48 h timepoint based on the slower clearance kinetics. All constructs were detectable in the tumor. For **Fab-IH18** and **Fab-IH18-PEG**, SPECT/CT images showed a clear tumor-background signal. As expected, high accumulation of **Fab-IH18** was observed in the kidneys. SPECT/CT images of **Fab-IH18-PEG** after 48 h were comparable to 24 h. The IVIS data show a clear signal at the tumor site, indicating that IVIS imaging can additionally be used to detect tracer accumulation in the tumor (Fig. 5). After the last scan, tumor and healthy tissues were collected for ex vivo biodistribution studies (Table S3). These results correlate with the findings from SPECT/CT and fluorescence imaging. Finally, we set out to visualize the localization of the multimodal imaging agent **mIgG1-IH18** on a tissue and cellular level. We analyzed tumor sections using immunohistochemistry (IHC) and performed autoradiography and fluorescence imaging of adjacent sections. This showed a large degree of co-localization of PD-L1 expression with the radioactive and fluorescent signal (Fig. 6A), with only a small amount of signal in PD-L1 negative regions. Overall, these data suggest in vivo tracer binding is target-specific and that the conjugates remain intact (Fig. 6A). Importantly, NIR fluorescence microscopy allowed us to determine the localization of the multimodal PD-L1 imaging agent and its co-localization with PD-L1 expression, even at cellular resolution (Fig. 6B, Additional file 1: Fig. S7).

## Discussion

While PD-L1 blockade yields durable responses in subgroups of cancer patients, many patients do not benefit from this treatment. As such, there is a clear need to better understand the biomarkers and dynamics of treatment response in order to improve patient stratification. To gain more insight into the fundamentals of anti-PD-L1 treatment, we developed multimodal, multiscale PD-L1 imaging agents to image PD-L1 drug distribution in vivo.

In this work we took advantage of the genetically engineered MIH5 derived hybridoma lines we created via our CRISPR/HDR approach to produce isotype switched mIgG1 chimeric PD-L1 targeting antibodies, as well as Fab fragments equipped with a sortag and a histag at the c-terminus of the heavy chain [27]. We synthesized a sortaggable peptide (**IH20**) carrying a DTPA metal chelator, as well as a cysteine and azidolysine for further functionalization. We attached a sulfo-Cy5 fluorophore as a proof of concept (**IH18**), but in principle any fluorescent and/or photodynamic dye could be attached. Sortase mediated chemoenzymatic conjugation enabled us to produce a panel of site-specific conjugates in good yields. The applicability of our CRISPR/HDR platform to any hybridoma, in combination with the modular conjugation strategy, makes our approach adaptable to a wide range of targets, as well as imaging modalities and other cargo molecules to produce molecularly defined conjugates in a reproducible manner.

Our in vivo results showed that isotype switching and site-specific modification of MIH5 aPD-L1 antibodies does not significantly change tumor uptake. However, hepatic accumulation was increased twofold for the mIgG1 PD-L1-srt-his antibody compared to the other antibodies, most likely caused by the presence of the Histag [46, 47]. Although previous studies showed that random DTPA conjugation via lysine side chains can influence affinity [24], in vivo pharmacokinetics [25, 48] and therapeutic index [49], we did not observe differences in blood clearance or tumor uptake for site-specifically versus randomly labeled mIgG1 aPD-L1 with DTPA or **IH20**. Finally, no statistical differences in biodistribution were observed for **IH20**- and **IH18**-conjugated chimeric mIgG1 aPD-L1, except for the spleen, where uptake of **mIgG1-IH18** was significantly higher, potentially caused by the apparent higher blood concentration 24 h p.i. Taken together, site-specific conjugation with **IH18** and **IH20** was feasible without major changes in the tumor targeting.

Side-by-side comparison of the different multimodal imaging formats showed that antigen specificity was retained for **mIgG1-IH18**, **Fab-IH18** and **Fab-IH18-PEG**. The IC_50_ value of **mIgG1-IH18** was in the same range as that of the WT MIH5 antibody. In accordance with literature, the Fab’s IC_50_ was 100-fold higher, due to its monovalency [27, 42, 50, 51]. Previously, it has been shown that non-site-specific PEGylation strongly decreases affinity [44], while site-specific PEGylation leads to a (largely) preserved affinity [42, 52–54]. The slight decrease in affinity for **Fab-IH18-PEG** could be caused by transient intramolecular blocking of the binding site by the long PEG chain, resulting in a slower on rate [55]. After labeling with ^111^In, all three site-specific multimodal imaging agents displayed target-specific binding and internalization (Additional file 1: Fig. S5b-d). In vivo, ^111^In-labeled conjugates accumulated in the tumor and other PD-L1-expressing tissues such as lymph nodes, brown fat and duodenum. Most notably, the rate of clearance was different for each of these three constructs, with **mIgG1-IH18** reaching its optimum TBR at 72 h. In contrast, **Fab-IH18** displayed a similar tumor uptake to **mIgG1-IH18** but a higher TBR already after 24 h. In translation to the clinic, this would mean faster consecutive imaging, more insight into the pharmacokinetics, as well as a shorter wait period between injection and imaging, making **Fab-IH18** superior to **mIgG1-IH18**. Compared to **Fab-IH18**, **Fab-IH18-PEG** showed increased tumor retention, which is likely caused by the increased circulation time: blood concentration is the driving force for initial diffusion into the tumor, where the antibody (fragment) can subsequently be retained by target binding [41].

SPECT/CT and IVIS imaging demonstrated non-invasive multimodal detection of the aPD-L1 imaging agents, correlating with the biodistribution results. Subsequent analysis of tumor sections showed a very strong co-localization between PD-L1 expression and the fluorescent and radioactive signals. However, especially for Fab PD-L1-IH18-PEG some additional tracer accumulation was observed in areas that were not positive for PD-L1 based on IHC. This could indicate a small degree of unspecific tracer uptake caused by the enhanced permeability and retention effect, or could be attributed to comparing adjacent sections. Together, these data indicate that in vivo tracer binding is largely target-specific and that our conjugates remain intact. Small antibody formats such as Fab fragments have been linked to improved tissue penetration in vitro [56] and due to their high diffusion rate [57], they have been hypothesized to have higher mobility in the extracellular matrix (ECM) of tumor cells [41]. Moreover, their small size causes less molecular crowding and they are therefore less likely to display the so-called “binding site barrier effect” [41]. However, we did not see any evidence that this applies to our multimodal imaging conjugates. The overall performance of our multimodal conjugates is comparable to previously published PD-L1 imaging agents [58, 59].

Contradicting correlations have been found between clinical whole-body PD-L1 PET imaging and treatment response [22, 23, 60, 61]. Our multimodal platform enabled us to image aPD-L1 tracer localization with cellular resolution with NIR fluorescence microscopy. This could provide valuable insight, because the cell type expressing PD-L1 has been linked to therapeutic outcome. For example, PD-L1 expressed by immune cells correlated with response for aPD-L1 therapy in some tumor types, whereas PD-L1 expressed by tumor cells did not [13, 14], while others have reported the reverse [15, 16]. Therefore, direct visualization of the cellular localization of the PD-L1 fraction which can be targeted with multimodal theranostic antibody-based imaging agents and hence antibody therapeutics could provide valuable insight into the correlation of PD-L1 expression with response to aPD-L1 therapy.

## Conclusion

We demonstrate that our modular platform allows for site-specific functionalization of aPD-L1 formats with peptides containing sulfo-Cy5 as a fluorophore and DTPA for radiolabeling, without significantly impacting the pharmacokinetic properties. We showed that fluorescent and radiolabeled **mIgG1-IH18**, **Fab-IH18** and **Fab-IH18-PEG** aPD-L1 display different pharmacokinetic properties, especially in terms of clearance. Our multimodal imaging agents allowed for whole-body SPECT/CT and IVIS imaging, as well as microscopy imaging, and we show that tracer accumulation is mainly target specific. Together, these data demonstrate that site-specific chemo-enzymatic conjugation of multiple imaging moieties to aPD-L1 antibodies is feasible and gives rise to molecularly defined multimodal PD-L1 imaging tools. Furthermore, the methodology described in our paper could be easily adapted for other targets. These agents can be employed to investigate immunotherapy in a preclinical setting, using whole-body imaging as well as analysis on tissue level and could potentially be translated for clinical applications.

## Materials and methods

### General methods and materials

Chemicals were used without further purification. Amino acids were purchased from Bachem (Bubendorf, Switzerland) or Novabiochem (EMD Chemicals, Gibbstown, USA), except for Fmoc-NH-PEG_3_-COOH (Iris Biotech, Marktredwitz, Germany). Solvents were obtained from ThermoFisher Scientific, Merck Millipore and Biosolve. LC–MS data were recorded on a Thermo Finnigan LCQ Fleet system, which consists of a Shimadzu LC-20A Prominence system (Shimadzu, ‘s-Hertogenbosch, The Netherlands) with a Gemini NX-C18 column, 150 × 2.1 mm, particle size 3 μm (Phenomenex, Utrecht, The Netherlands) with PDA detector coupled to a Thermo LCQ Fleet mass spectrometer. For LC–MS analysis, an acetonitrile/water gradient was used of 5–100% in 60 min, flow rate 0.2 mL/min. For peptide purification, preparative HPLC was performed on a Shimadzu LC-20A Prominence system (Shimadzu, ‘s-Hertogenbosch, The Netherlands) equipped with a Gemini NX-C18 column, 150 × 21.20 mm, particle size 10 μm (Phenomenex, Utrecht, The Netherlands) or on a Waters HPLC system equipped with an XBridge Prep-C18 column, particle size 5 µm, 150 × 30 mm and an Equity QDa Mass Detector. On the Shimadzu, the gradient used was acetonitrile/water 5–40% in 35 min, flow rate 10 mL/min. On the Waters HPLC system, the acetonitrile/water gradient was 5–25% in 10 min, flow rate 40 mL/min. Analytical HPLC was performed on a Shimadzu LC-20A Prominence system (Shimadzu, ‘s-Hertogenbosch, The Netherlands) equipped with a Gemini NX-C18 column, 150 × 3 mm, particle size 3 µm (Phenomenex, Utrecht, The Netherlands). For both the analytical and preparative HPLC, peptides were monitored at 254 and 215 nm.

Fluorescence analysis was carried out using the Typhoon Trio Variable Mode Imager System from GE Healthcare (Eindhoven, The Netherlands). SDS-PAGE coomassie analysis was performed using a Bio-rad ChemiDoc XRS + system. For size-exclusion chromatography (SEC) and cation-exchange chromatography (CEX), a Bio-rad NGC Quest Plus System with 4-wave UV–Vis detector and a 10 mL/min pump (Bio-rad, Veenendaal, The Netherlands). For SEC, The NGC system was equipped with a Superdex 75 Increase 10/300 GL Size-exclusion column (GE Healthcare, Eindhoven, The Netherlands). For CEX, a 1 mL HiTrap SP FF (GE Lifesciences, 17505401) cation exchange column was used on the same NGC Quest Plus Bio-Rad system, operating at 0.5 mL/min. Antibody concentration was determined using the NanoDrop 2000c (Thermo Fisher Scientific). FACS measurements were performed on FACSVerse (BD Biosciences), and analysis was performed using FlowJo V10 software.

### Cell culture

Previously, MIH5 hybridoma cells were engineered to express mIgG1, mIgG2a_silent_ or Fab fragments against PD-L1 with a Sortag and a Histag at the C-terminus of the heavy chain(s) (plasmids available at www.addgene.org/, ID: 124802, 124807 and 124810) [27]. Other cell lines that were used throughout this study were 4T1 (ATCC CRL-2539) and Renca (ATCC CRL-2947). All cells were cultured in RPMI-1640 (Gibco, 11875-093, Thermo Fisher Scientific) supplemented with 10% heat-inactivated fetal calf serum (FCS), 2 mM UltraGlutamine (BE16-605E/U1, Lonza) and 1 × antibiotic–antimycotic (15240-062, Thermo Fisher Scientific). In addition, Hybridoma medium contained 50 μM Gibco 2-mercaptoethanol (2-ME) (21985-023, Thermo Fisher Scientific). Renca cell medium additionally contained 0.1 mM non-essential amino acids (NEAA) (11140-035, Thermo Fisher Scientific) and 1 mM Sodium Pyruvate (Gibco, 11360-070), Thermo Fisher Scientific). (Semi-) adherent cells were washed with phosphate-buffered saline (PBS) (Fresenius Kabi) and detached by incubation with 0.025% trypsin and 0.01% ethylenediaminetetraacetic acid (EDTA) in PBS (TE) (Thermo Fisher, R001100) for 10 min. at 37 °C, or by using a cell scraper.

### Production of antibody conjugates

#### Production and isolation of sortaggable antibodies

After expansion, Hybridoma cells were seeded in a CELLine Disposable Bioreactor (Corning, 353137). The medium compartment contained 900 mL Hybridoma medium with 1% FBS, the cell compartment contained 15 mL Hybridoma medium with 10% FBS. Medium was harvested from cell compartment every 7 days and separated from the cells by centrifugation (5 min, 1500 rpm), filtered through a 20 µm filter (Whatman) and stored at − 20 °C until the moment of antibody purification. Pellet was resuspended in 30 mL medium and live cells were separated from dead cells by using Ficoll density centrifugation (Lymphoprep; Axis-Shield PoC AS, Oslo, Norway). Subsequently, 30 million live cells were reseeded into the CELLine bioreactor. For each flask, multiple seeding—harvesting cycles were performed. For antibody purification, the supernatant was thawed and incubated for 20 min at room temperature (RT) with 1–2 mL Ni–NTA beads (Qiagen, 30210). Subsequently, the suspension was transferred to an Econo-Pac Chromatography Column (Bio-Rad, 7321010). The column was washed with 10 column volumes (CV) of wash buffer (50 mM NaH_2_PO_4_.H_2_O, 300 mM NaCl, 20 mM imidazole, 0.05% Tween 20, pH 8.0), 100 CV of 0.1% Triton X-114 (Merck, 9036-19-5) in sterile PBS at 4 °C, and 20 CV sterile PBS. These three steps were repeated twice. Finally, the antibody was eluted with elution buffer (50 mM NaH_2_PO_4_.H_2_O, 300 mM NaCl, 250 mM imidazole, 0.05% Tween 20, pH 8.0). The resulting elution fractions were concentrated with Amicon Ultra-15 Centrifugal 10 kDa MWCO filter units (Merck, Z717185) for Fab fragments and 50 kDa MWCO filter units for monoclonal antibodies. Buffer exchange was performed using sortase buffer (50 mM Tris, 150 mM NaCl, pH 7.5). Antibody concentration was determined using the NanoDrop 2000c (Thermo Fisher Scientific) using the protein program and dividing by 1.4 for monoclonal antibodies and by 1.35 for a Fab fragment. Protein purity was assessed under reducing conditions using SDS-PAGE gel electrophoresis (12% acrylamide) and Sypro Ruby Protein Gel stain (S12000, Thermo Fisher Scientific). Typical yields per week were around 2–2.5 mg for Fab and 1.5–2 mg for mIgG1.

#### Peptide synthesis

The backbone of multimodal imaging peptides **IH20** and **IH18** (Fig. 1b) was synthesized on Rink resin (500 mg, 0.29 mmol) using standard Fmoc-based Solid-Phase Peptide Synthesis (SPPS). Coupling and deprotection steps were followed to completion using a Kaiser test. For each coupling step, 3 eq. of amino acid (AA) was used. For most coupling steps, 3.3 eq. of 1 M DIPCDI and 3.6 eq. of 1 M HOBt in DMF were used. Coupling steps were incubated for 45 min to overnight. For coupling of H_2_N-PEG_3_-COOH, 1.5 eq. HATU and 3 eq. DIPEA were added and this mixture was agitated overnight. Upon completion of each coupling step, as indicated by the Kaiser test, the resin was washed three times with DMF and the remaining free amines were capped using a 3:2 mixture of Acetic anhydride and pyridine in DMF. After capping, the resin was washed three times again with DMF. Subsequently, the Fmoc group on the last AA in the chain was removed by incubating the resin with 20% piperidine for 20 min. After each deprotection, the next AA was coupled as described. For the N-terminal glycine, Boc-protected glycine was used instead of Fmoc-protected to allow for easy removal of all protective groups when cleaving the peptide from the resin. Upon completion of the backbone, the resin was washed subsequently with DMF (3×), DCM (3×) and diethyl ether (3×). Peptide on resin was then dried and stored at − 20 °C.

To allow the chelation of radionuclides, the chelator DTPA was attached to the lysine side chain of the peptide. The resin (300 mg, 120 µmol) was swelled in DCM, before multiple cycles of treatment with 1.2% TFA in DCM for 2 min (optimal deprotection after 15 cycles), After which the resin was washed extensively with DCM and subsequently with DMSO. Then, S-2-(4-Isothiocyanatobenzyl)-diethylenetriamine pentaacetic acid (p-SCN-Bn-DTPA, Macrocyclics, B-305) (236 mg, 360 µmol, 3 eq.) was dissolved in DMSO and DIPEA (0.73 mL, 4.20 mmol, 35 eq.) was added. This mixture was agitated with the resin overnight at RT. Finally, the peptide was fully deprotected and cleaved off the resin by incubating with a 92.5/2.5/2.5/2.5 mixture of TFA/H_2_O/TIS/Thioanisole for 2–3 h. The peptide was precipitated in ice cold diethyl ether and air-dried. After drying, the resulting off-white solid was dissolved in 80/20 H_2_O/ACN and lyophilized.

The resulting **IH20** peptide was purified using RP-HPLC (10–40% ACN/H_2_O in 35 min), and analyzed using LC–MS. HPLC rt: 20.67. LC–MS(ESI +): *m/z* calcd for C_52_H_84_N_16_O_20_S_2_^2+^ ([M + 2H]^2+^) 659.28, found 659.30. C_52_H_84_N_16_O_20_S_2_^3+^ ([M + 3H]^3+^): 439.85, found 439.94. After purification and lyophilization, **IH20** was obtained as a white powder (yield 43%).

To yield **IH18** by modification of **IH20** with sulfo-Cy5, **IH20** (10.8 mg, 7.54 µmol) was dissolved in DMF (60 mg/mL) and mixed with 1.1 eq. (6.7 mg, 8.30 µg) sulfo-cyanine-5-maleimide (Lumiprobe, 13380) in PBS (pH 6.9, 9.5 mg/mL). This mixture was agitated overnight at RT. The resulting **IH18** peptide was purified using RP-HPLC (5–40% ACN/H_2_O in 35 min), and analyzed using LC–MS. HPLC rt: 27.27. LC–MS (ESI +) *m/z* calcd for C_90_H_128_N_20_O_29_S_4_^2+^ ([M + 2H]^2+^) 1041.40, found 1041.80. C_90_H_128_N_20_O_29_S_4_^3+^ ([M + 3H]^3+^): 694.60 found: 694.84. After lyophilization, **IH18** was obtained as a blue powder (10.6 mg, 4.83 µmol, yield 64%).

#### Site-specific enzymatic conjugation

After optimization, batch sortagging was carried out using 0.5–1.0 mg of antibody (fragment) and 25 eq of **IH18** or **IH20** for mIgG1 PD-L1 and 50 eq of **IH18** or **IH20** for Fab PD-L1. After termination of the reaction with EDTA (final concentration of 10 mM), the reaction mixture was incubated with 200 µl Ni–NTA beads for 20 min at RT, in order to remove unreacted antibody and Sortase. Beads were separated from the reaction mixture using empty spin columns (Jena Bioscience, AC-552-25), and washed twice with wash buffer (50 mM NaH_2_PO_4_.H_2_O, 300 mM NaCl, 20 mM imidazole, 0.05% Tween 20, pH 8.0) and twice with PBS. Reaction mixture and wash fractions were combined and purified using size-exclusion chromatography (SEC), with 1 mM EDTA in PBS as buffer (10 mL/min). Fractions containing the product were combined and concentrated using Amicon Ultra-15 Centrifugal Filter Units, MWCO 10 kDa (Merck, Z717185). Buffer exchange was performed using sterile PBS. Antibody concentration was determined on NanoDrop, using the UV–vis program and measuring at 280 nm for **IH20** and at 280 and 646 nm for **IH18**. Protein purity was assessed under reducing conditions using SDS-PAGE gel electrophoresis (12% acrylamide), comparing starting material, product and product clicked with 5 kDa mPEG-DBCO to verify functionalization of all heavy chains. Per construct, several batches were produced and these batches were combined before performing in vitro and in vivo experiments to guarantee batch uniformity. Quantification of protein purity was achieved using densitometry. Densitometry was performed in ImageJ, and defined as (product bands − background)/(non-product bands − background) × 100%. Purity mIgG1 PD-L1-IH18 = 85%, Fab PD-L1-IH18 = 96%.

#### PEGylation and purification of FabPD-L1-IH18

Fab PD-L1-IH18 was conjugated to 20 kDa PEG by adding 10 Eq. (94.0 nmol) of mPEG-DBCO (Click Chemistry Agents, A120) in PBS (3.33 mM) directly to Fab PD-L1-IH18 in PBS (470 µg, 9.40 nmol, 3.0 mg/mL). This mixture was incubated for 90 min at 37 °C, buffer exchange was performed with 0.02 M acetate buffer (pH 4.5, buffer A) using Amicon Ultra-4 Centrifugal Filter Units, MWCO 10 kDa (Merck, UFC801024) and subsequently purified using a 1 mL HiTrap SP FF (GE Lifesciences, 17505401) cation exchange column on an NGC Quest Plus Bio-Rad system operating at 0.5 mL/min. After equilibrating with buffer A for 8 column volumes, a linear gradient of 0.5 M NaCl in buffer A was applied (0–0.5 M NaCl in 30 column volumes). PEGylated Fab PD-L1-IH18 were typically eluted after about 8 column volumes (0.15 M NaCl). Fractions containing the purified product were concentrated using Amicon Ultra-4 Centrifugal 10 kDa MWCO filter units (Merck, UFC 801024) and sterile PBS as a buffer, and analyzed by 12% SDS-PAGE. The concentration was determined using Nanodrop. This method was verified as reliable by use of a BCA assay (Pierce BCA Protein Assay Kit, ThermoFisher Scientific). Quantification of protein purity was achieved by performing densitometry, as described above. Purity Fab PD-L1-IH18-PEG_20kDa_ = 89%.

#### Random conjugation of DTPA to PD-L1 antibodies

To prevent contaminants from disturbing radiolabeling, 1 mg of mIgG1 PD-L1-srt-his and rIgG2a WT antibodies were dialyzed against 5 L sterile PBS (metal-free) using Slide-a-Lyzer Cassettes (ThermoFisher Scientific). Subsequently, a 15 eq. of S-2-(4-Isothiocyanatobenzyl)-diethylenetriamine pentaacetic acid (p-SCN-Bn-DTPA, Macrocyclics, B-305) and 1/10 reaction volume 1 M NaHCO_3_ in PBS, pH 5.5 were added to each antibody and incubated for 1 h at RT. Non-conjugated p-SCN-DTPA was removed from the reaction mixture by dialysis against 5 L 0.25 M NH_4_Ac (metal-free, pH 5.5) using Slide-a-Lyzer Cassettes (ThermoFisher Scientific). After dialysis, concentration in all samples was determined via spectrophotometer.

### Radiolabeling

Antibody-IH18 conjugates were incubated with ^111^In (Mallinckrodt BV) in 0.5 M MES buffer (pH 5.4) for 20 min at room temperature under metal-free conditions as described previously [62]. Non-chelated ^111^In was complexed by adding 50 mM EDTA to a final concentration of 5 mM. Labeling efficiency was determined using thin-layer chromatography on silica gel chromatography strips (Agilent Technologies), using 0.1 M citrate buffer (Sigma-Aldrich, pH 6.0) as mobile phase. Samples with a labeling efficiency below 95% were purified using a PD-10 column (GE Healthcare, 17-0851-01) eluted with PBS. Radiochemical purity of ^111^In-labeled antibody-IH18 conjugates exceeded 95% in all experiments.

### In vitro characterization

#### IC_50_ of multimodal PD-L1 imaging conjugates

30,000 Renca cells were seeded per well in a 96-well V-bottom plate, and stained with 30 μl of mIgG1 PD-L1-IH18, Fab PD-L1-IH18, Fab PD-L1-IH18-PEG_20kDa_ or a mIgG1 isotype control (BioLegend, 400102). Concentrations of these constructs ranged from 0.1 pM to 1000 nM for antibodies and 0.4 pM to 6000 nM for Fabs. After 20 min of incubation at 4 °C, 30 μL of commercially available MIH5 PD-L1-PE (ThermoFisher Scientific, 12-5982-82) was added (final concentration 6.7 nM). After another 30 min of incubation at 4 °C, cells were washed and resuspended in PBA and measured on FACS. The IC_50_ was defined as the antibody-conjugate concentration that was required to inhibit binding of the commercially available fluorescently-labeled antibody by 50%.

#### Internalization kinetics

Renca cells were cultured to confluency in 6 wells plates and incubated for 1, 3 or 24 h with 105 pM radiolabeled mIgG1, 323 pM Fab or 86 pM PEGylated Fab in RPMI-1640 containing 1% BSA at 37 °C in a humidified atmosphere with 5% CO_2_. Non-specific binding and uptake were determined by co-incubation with 16 nM unlabeled WT PD-L1 (rIgG2a). After incubation, cells were washed and incubated with acid wash buffer (0.1 M AcOH, 0.15 M NaCl, pH 2.8) for 10 min at 37 °C to remove the membrane-bound fraction of the antibody conjugates. Cells were washed with PBS, and the acid wash and PBS were combined into one fraction containing the membrane-bound activity. Finally, cells containing the internalized activity were lysed with 0.1 M NaOH and harvested. Samples were measured in a gamma counter (Wizard^2^, Perkin-Elmer, Boston MA) to determine the internalized and membrane-bound activity.

### Animal studies

All experiments were performed in accordance with the revised Dutch Act on Animal Experimentation (2014) approved by the central authority for scientific procedures on animals and animal welfare body of the Radboud University, Nijmegen. Mice were housed in individually ventilated cages with a filter top (Blue line IVC, Tecniplast, West Chester, USA) under pathogen-free conditions with cage enrichment present (abundant bedding material and transparent dome), and were fed and watered ad libitum. Biotechnicians were blinded to all groups and compounds administered. Studies were performed with 6–8 weeks old BALB/c mice (Janvier, le Genest-Saint-Isle, France). In all experiments mice received 1 × 10^6^ 4T1 cells (ATCC) by injection into the mammary fat pad. Mice were block-randomized across the experimental groups based on tumor size. Experiments started when average tumor size reached 0.3 cm^3^.

#### Biodistribution study

For each construct, 3 groups of 5 mice received an intravenous injection of 0.4 pmol ^111^In-labelled tracer (0.4 MBq, 200 µL). At 1, 2 and 24 h after injection with Fab PD-L1-IH18 or Fab PD-L1-IH18-PEG_20kDa_, or 4, 24 and 72 h for mIgG1, mice were sacrificed using CO_2_/O_2_-asphyxiation. To determine the biodistribution of each construct, tumor and normal tissues (blood, lymph node, muscle, lung, spleen, thymus, kidney, liver, duodenum, colon, brown fat, bone marrow and bone) were harvested, weighed and measured in a gamma counter, as well as 1% standards of the injected dose. Values are reported as percentage injected dose per gram (%ID/g).

#### SPECT/CT imaging

For each construct, mice (n = 5/group) received 0.4 pmol ^111^In-labelled tracer (8 MBq, 200 µL). For Fab, scans were acquired at 4 h and 24 h post-injection (p.i.), for Fab-PEG at 4 h, 24 h and 48 h, and the full length antibodies were imaged at 24 and 72 h p.i.. Images were acquired for 45 min under general anesthesia (isoflurane in 100% oxygen, 5% for induction, 2% maintenance) with the U-SPECT-II/CT (MILabs, Utrecht, The Netherlands) using a 1.0 mm diameter pinhole mouse high sensitivity collimator, followed by CT scan (615 µA, 65 kV) for anatomical reference. Scans were reconstructed with MILabs reconstruction software using a 16-subset expectation maximization algorithm, with a isotropic voxel size of 0.2 mm and 1 iteration. SPECT/CT scans were analyzed and maximum intensity projections (MIP) were created using Inveon Research Workplace software (Siemens).

### Autoradiography & fluorescence imaging of tissue sections

Snap frozen, unstained tumor section (10 µm) were scanned on a fluorescence flat-bed scanner at pixel resolution of 4 µm and with an excitation/filter setting of 690/700 nm, quality setting ‘high’ (Odyssey CLx, LICOR) and subsequent sections were exposed to a Fujifilm BAS cassette 2025 (Fuji Photo Film) for 3 days. Phosphor luminescent plates were scanned using a phosphor imager (Typhoon FLA 7000; GE) at a pixel size of 25 × 25 µm. Images were acquired with Aida Image software.

### Immunohistochemistry

PD-L1 expression in tumors was determined by chromogenic detection using fresh frozen tissue sections fixed in PBS 45% acetone 10% formalin. In short, sections were pre-incubated with 10% normal rabbit serum, endogenous biotin and avidin were blocked, and endogenous peroxidase activity was blocked with 3% H2O2. Subsequently, anti–mPD-L1 (0.4 μg/mL, AF1019, R&D systems) was applied and tissues were incubated overnight at 4 °C. Next, sections were incubated with biotinylated rabbit anti-goat IgG (3.75 μg/mL E0466, DAKO) followed by incubation with avidin–biotin-enzyme complexes (dilution 1:50, Vector Laboratories, Burlingame, CA). Finally, 3’,3’-diaminobenzidine (DAB) was used to develop the tumor sections prior to mounting slides.

Furthermore, fluorescence detection was performed to co-localize mIgG1 PD-L1-IH18 with PD-L1 expression. Fresh frozen tissue sections were fixed using buffered formalin-acetone fixative and pre-incubated with 10% normal donkey serum or 10% normal goat serum. Subsequently, slides were incubated overnight at 4 °C with anti-mPD-L1 (10 µg/mL, AF1019 R&D biosystems) or isotype control goat IgG (10 µg/mL, 005–000-003, Jackson Immunoresearch). Next, donkey-anti-goat-Alexa488 (2.5 µg/mL in glycerol, A11055, Thermo Fisher) was applied for 30 min and DAPI (0.5 µg/mL, D1306, Invitrogen) for 2 min at RT. Sections were mounted with fluoromount (F4680, DAKO) and fluorescence imaging was performed with a Leica DMI6000 epi-fluorescence microscope fitted with a 63 × 1.4 NA oil immersion objective.

### Statistical analyses

Statistical analyses were performed using GraphPad Prism version 5.03 (San Diego, CA) for Windows. Data are presented as mean ± standard deviation. Differences in uptake of the radiolabeled tracers were tested for significance using a one-way ANOVA with a Bonferroni test. A p-value below 0.05 was considered significant.

## Supplementary Information

**Additional file 1.** Supplementary figures and tables.

## Data Availability

The dataset used and/or analysed during the current study are available from the corresponding author on reasonable request.
