# Correction to: Multiscale imaging of therapeutic anti-PD-L1 antibody localization using molecularly defined imaging agents Iris

**DOI:** 10.1186/s12951-022-01306-y

**Published:** 2022-05-14

**Authors:** Iris M. Hagemans, Peter J. Wierstra, Kas Steuten, Janneke D. M. Molkenboer-Kuenen, Duco van Dalen, Martin ter Beest, Johan M. S. van der Schoot, Olga Ilina, Martin Gotthardt, Carl G. Figdor, Ferenc A. Scheeren, Sandra Heskamp, Martijn Verdoes

**Affiliations:** 1 Department of Tumor Immunology, Radboud Institute for Molecular Life Sciences, Radboud University Medical Center, Nijmegen, The Netherlands; 2 Institute for Chemical Immunology, Nijmegen, The Netherlands; 3 Department of Medical Imaging, Nuclear Medicine, Radboud Institute for Molecular Life Sciences, Radboud University Medical Center, Nijmegen, The Netherlands; 4 Division of Immunotherapy, Oncode Institute, Radboud University Medical Center, Nijmegen, The Netherlands; 5 Department of Dermatology, Leiden University Medical Centre, Leiden, The Netherlands

## Correction to: Journal of Nanobiotechnology (2022) 20:64 https://doi.org/10.1186/s12951-022-01272-5

Following publication of the original article [1], the authors identified an error in Fig. 1 and Fig. 4. The correct figures are given in this correction article.

The error was in the “R-groups” (R1 and R2) of the molecules IH18 and IH20 in Fig. 1b and in Additional file 1: Figure S1. R1 and R2 were accidently swapped. In Fig. 4d the error was in the depiction of the tumor/blood ratio. During transfer of the data to this bar graph the data got unintentionally transformed.

**Fig. 1:**
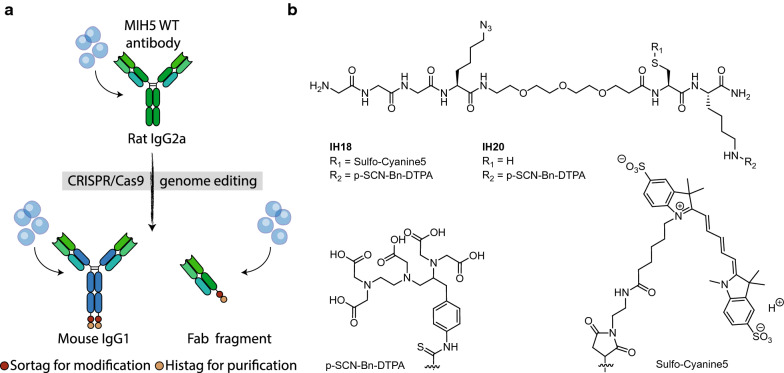
Molecular toolbox of site-specific functionalizable anti-PD-L1 antibody formats and imaging peptides.** A** Application of the CRISPR/HDR strategy to anti-PD-L1 hybridoma MIH5 created a sortaggable Fab fragment and chimeric mouse IgG1 monoclonal antibody against PD-L1. **B** Molecular structure of imaging peptides IH20 and IH18

**Fig. 4:**
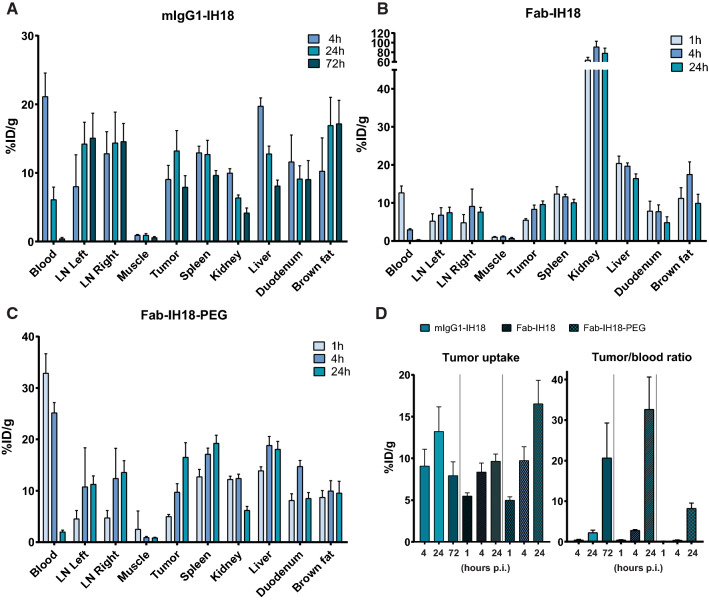
Biodistribution results of anti-PD-L1 multimodal imaging tools. Biodistribution is shown at several time points after injection (p.i.) of ^111^In-labeled **A** mIgG1-IH18, **B** Fab-IH18 or **C** Fab-IH18-PEG. **D** Tumor uptake values (left graph) and tumor/blood ratios (right graph) of the three constructs are compared directly at each time point. Data are shown as mean ± SD, n = 5

## Supplementary Information

**Additional file 1.** Supplementary figures and tables.
