# Supplementary material for: Correction to: Multiscale imaging of therapeutic anti-PD-L1 antibody localization using molecularly defined imaging agents Iris
Source: J Nanobiotechnology. 2022 May 14;20:229. doi: 10.1186/s12951-022-01306-y (PMC9107661; doi:10.1186/s12951-022-01306-y)
Supplement: Supplementary file 1 — Additional file 1. Supplementary figures and tables. [file 12951_2022_1306_MOESM1_ESM.pdf]

Supplementary figures and tables for:

Multiscale imaging of therapeutic anti-PD-L1 antibody localization using molecularly defined imaging agents.

Iris M. Hagemans[#,1,2], Peter J. Wierstra[#,3], Kas Steuten[1,2], Janneke D.M. Molkenboer-Kuenen[3], Duco van Dalen[1,2], Martin ter Beest[1], Johan M.S. van der Schoot[1], Olga Ilina[1,2], Martin Gotthardt[3], Carl G. Figdor[1,2,4], Ferenc A. Scheeren[5], Sandra Heskamp[#,3,*], Martijn Verdoes[#,1,2,*].

[#]*these authors contributed equally*

*Corresponding authors: Martijn.Verdoes@Radboudumc.nl, Sandra.Heskamp@Radboudumc.nl.

1. Department of Tumor Immunology, Radboud Institute for Molecular Life Sciences, Radboud University Medical Center, Nijmegen, Netherlands

2. Institute for Chemical Immunology, Nijmegen, Netherlands

3. Department of Medical Imaging, Nuclear Medicine, Radboud University Medical Center, Radboud Institute for Molecular Life Sciences, Nijmegen, The Netherlands.

4. Division of Immunotherapy, Oncode Institute, Radboud University Medical Center, Nijmegen, Netherlands

5. Department of Dermatology, Leiden University Medical Centre, Leiden, The Netherlands

Figure S1: Synthetic route for IH20 and IH18.

Figure S2: LC-MS analysis of IH20 and IH18.

Figure S3: Purification of multimodal imaging constructs.

Figure S4: SDS-PAGE analysis of multimodal PD-L1 imaging tools.

Figure S5: *In vitro* analysis of constructs.

Figure S6: Biodistribution results of multimodal PD-L1 imaging tools for SPECT.

Figure S7: Fluorescence microscopy of tumor sections for mIgG1-IH18 localization.

Table S1: Biodistribution results of anti-PD-L1 monoclonal antibody panel.

Table S2: Biodistribution results of multimodal PD-L1 imaging tools.

Table S3: Biodistribution results of PD-L1 imaging study.

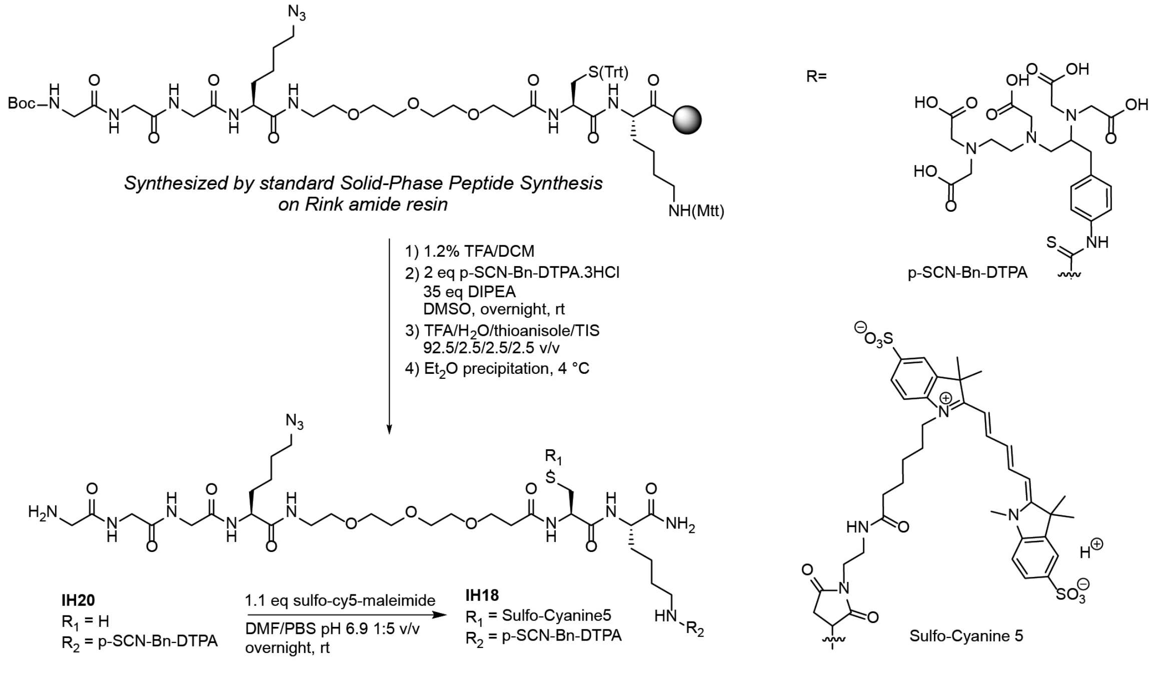

Figure S1. Synthetic route for IH20 and IH18. The peptide backbone was synthesized according to Fmoc-based SPPS. The lysine is selectively deprotected on resin and reacted with p-SCN-Bn-DTPA. After cleavage from the resin and precipitation in ice cold ether, the peptide was purified to yield IH20. Conversion to IH18 consists of a reaction between the cysteine and sulfo-Cy5-maleimide and subsequent purification.

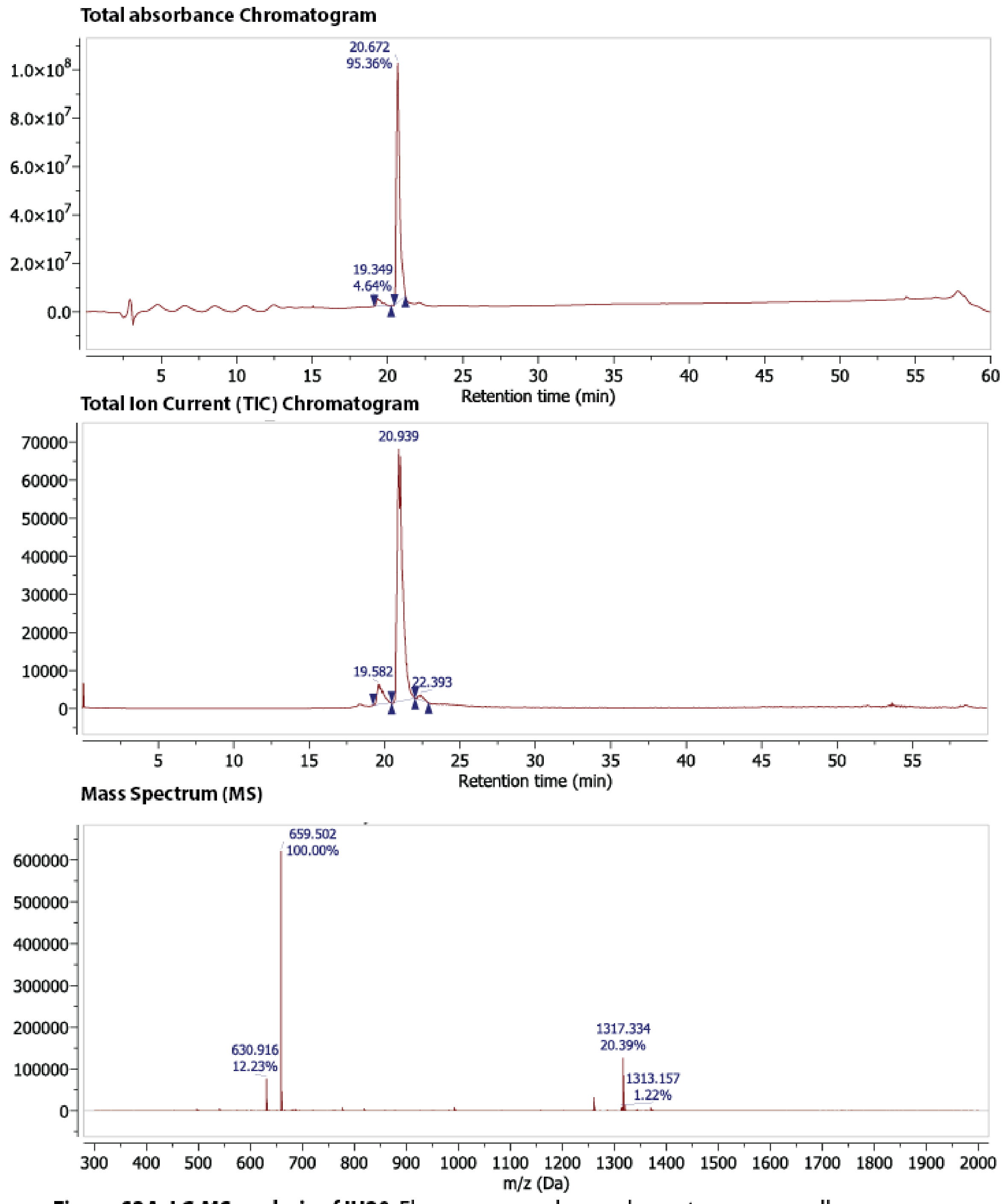

Figure S2A. LC-MS analysis of IH20. Fluorescence and mass chromatograms as well as mass spectrum for IH20.

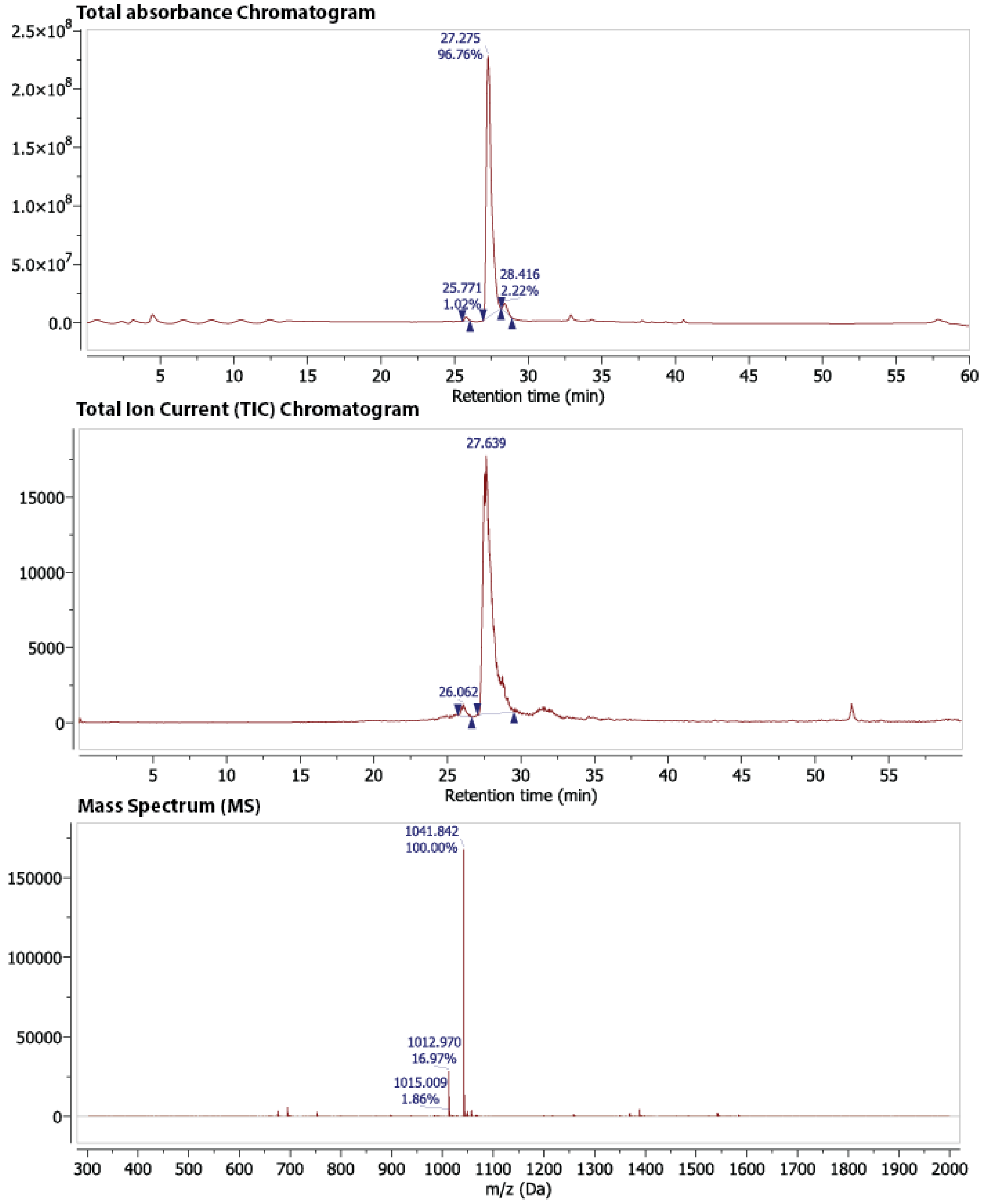

Figure S2B. LC-MS analysis of IH18. Fluorescence and mass chromatograms as well as mass spectrum for IH18.

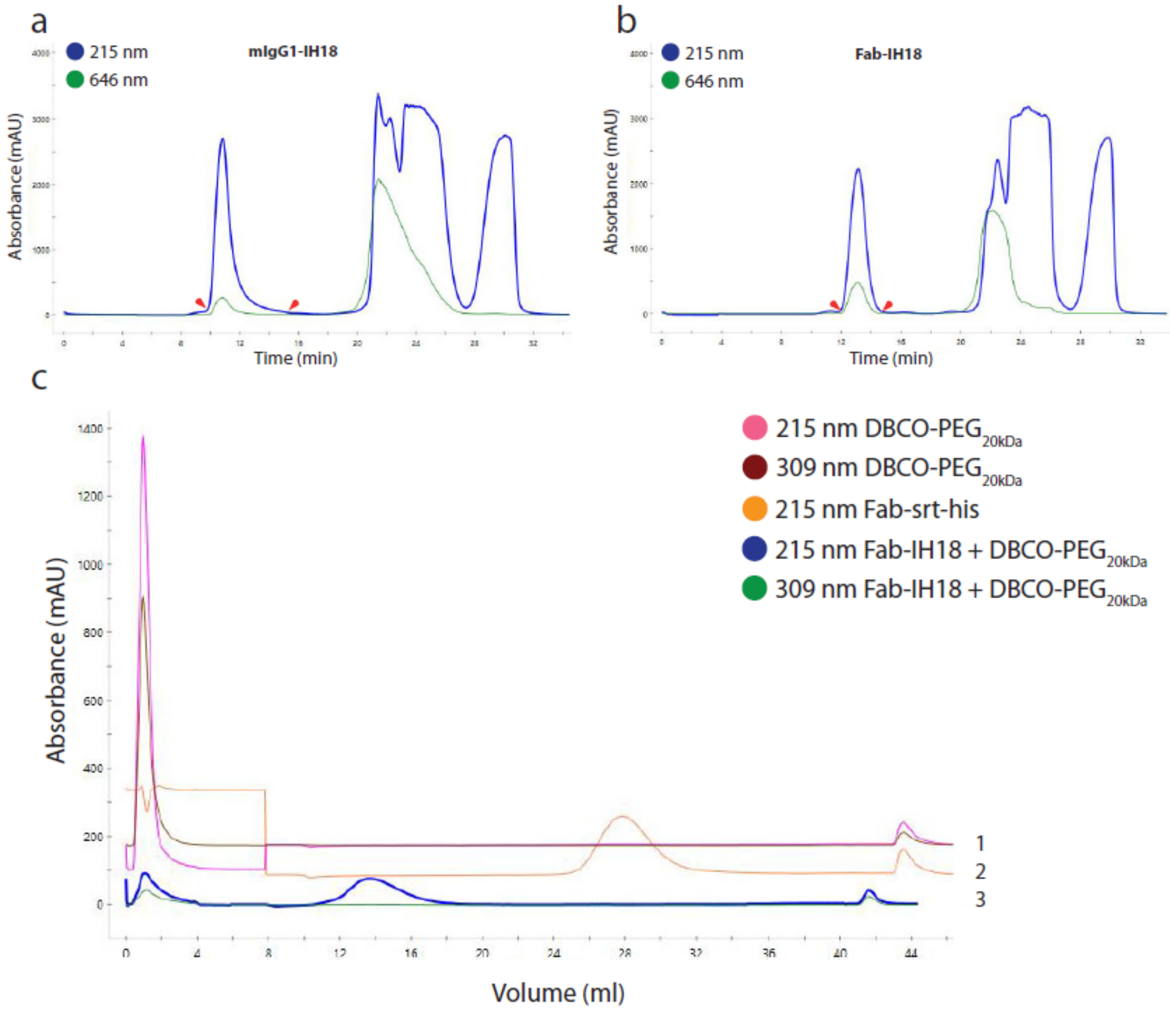

Figure S3. Purification of multimodal imaging constructs. Purification after sortagging consisted of incubation with NiNTA beads to remove starting material and sortase and size-exclusion chromatography to separate product from free peptide. Chromatograms are shown for **a)** mIgG1 and **b)** Fab PD-L1-IH18. Red arrows indicate where product elution begins and ends. **c)** For generation of the PEGylated Fab fragment, cation exchange chromatography was performed after a SPAAC reaction with 20kDa mPEG-DBCO. 1) 20kDa DBCO-mPEG alone, 2) Fab PD-L1-srt-his alone and 3) Reaction mixture after SPAAC.

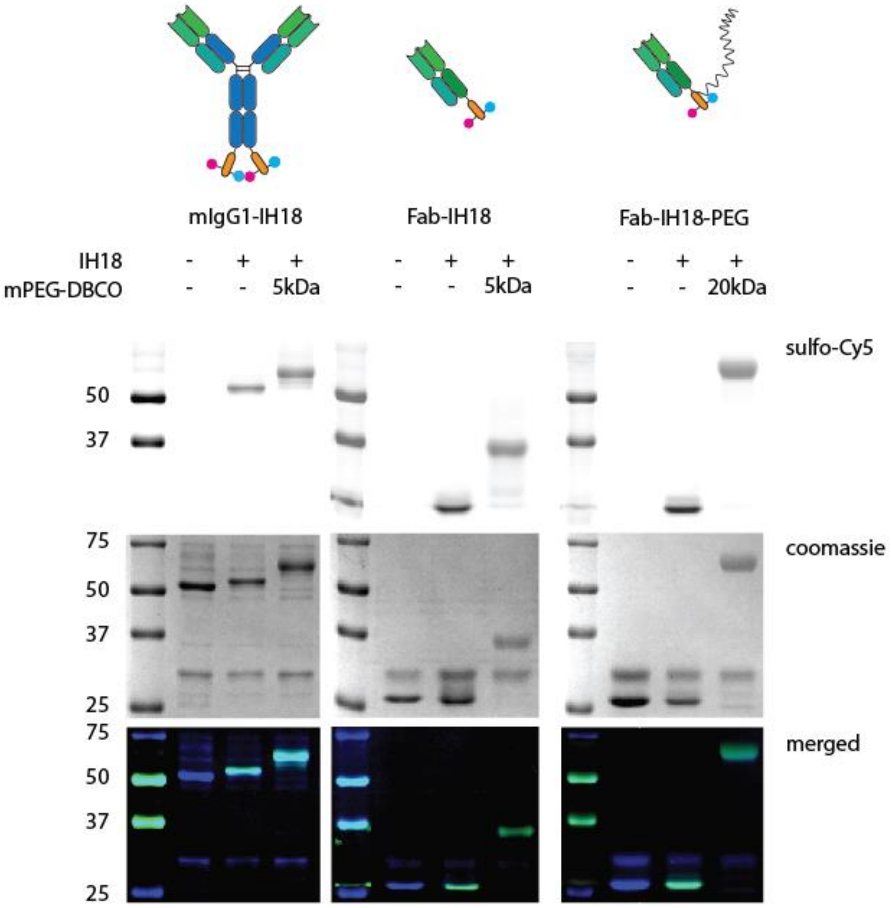

Figure S4. SDS-PAGE analysis of multimodal PD-L1 imaging tools. SDS-PAGE analysis of mIgG1, Fab and Fab-PEG$_{20kDa}$ PD-L1-IH18. After purification using NiNTA beads and SEC, an analytical fraction of mIgG1 and Fab PD-L1-IH18 was reacted with 5kDa mPEG-DBCO to analyze purity and azide functionality. This resulted in the near-quantitative conversion to a fluorescent product of higher molecular weight. Fab-PEG20kDa was produced through large-scale reaction of Fab PD-L1-IH18 with 20kDa mPEG-DBCO and subsequently purified using cation exchange chromatography.

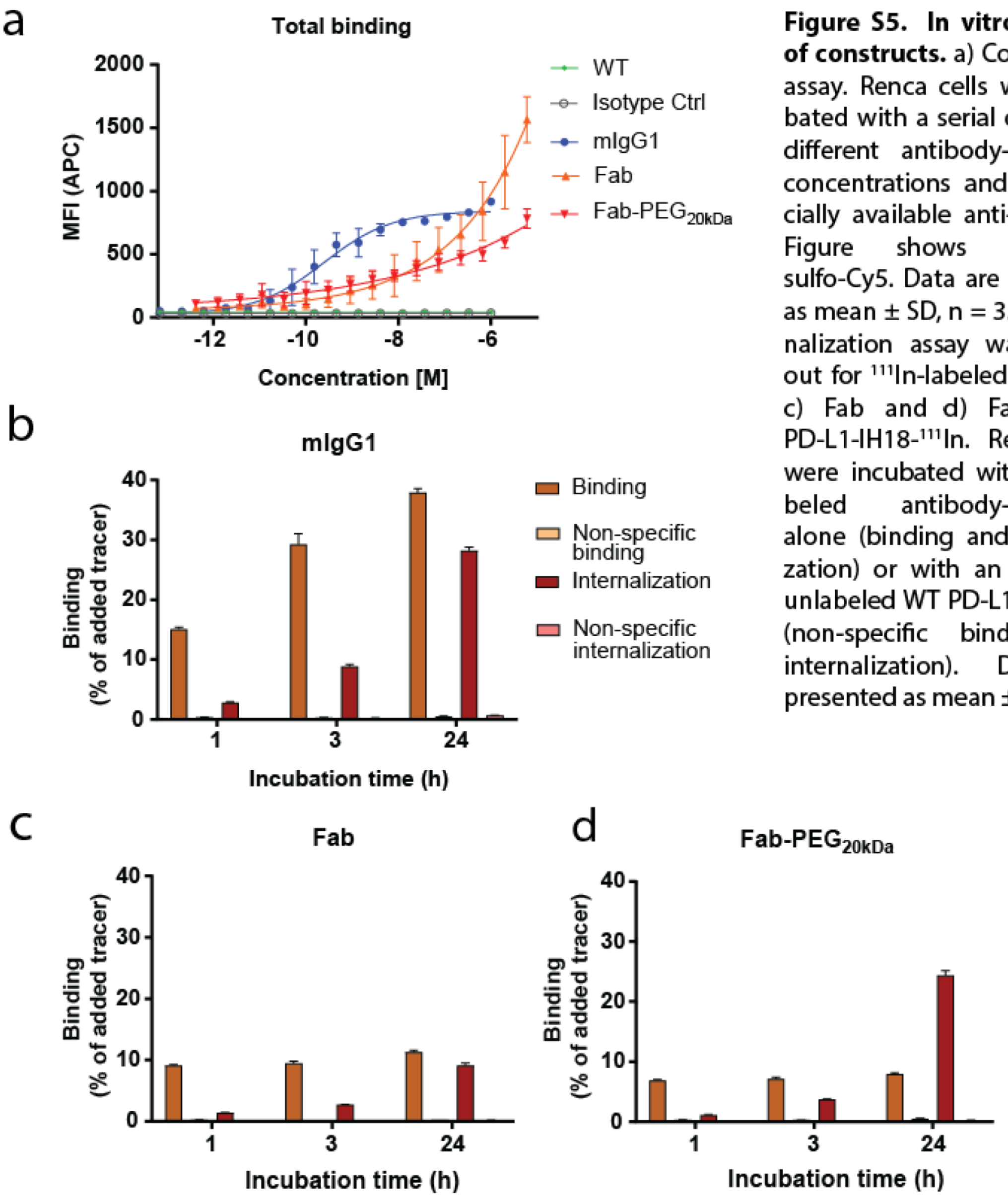

Figure S5. In vitro analysis of constructs. a) Competition assay. Renca cells were incubated with a serial dilution of different antibody-conjugate concentrations and commercially available anti-PD-L1-PE. Figure shows MFI for sulfo-Cy5. Data are presented as mean ± SD, n = 3. An internalization assay was carried out for ^{111}In-labeled b) mIgG1 c) Fab and d) Fab-PEG$_{20kDa}$ PD-L1-IH18-^{111}In. Renca cells were incubated with radiolabeled antibody-conjugate alone (binding and internalization) or with an excess of unlabeled WT PD-L1 antibody (non-specific binding and internalization). Data are presented as mean ± SD.

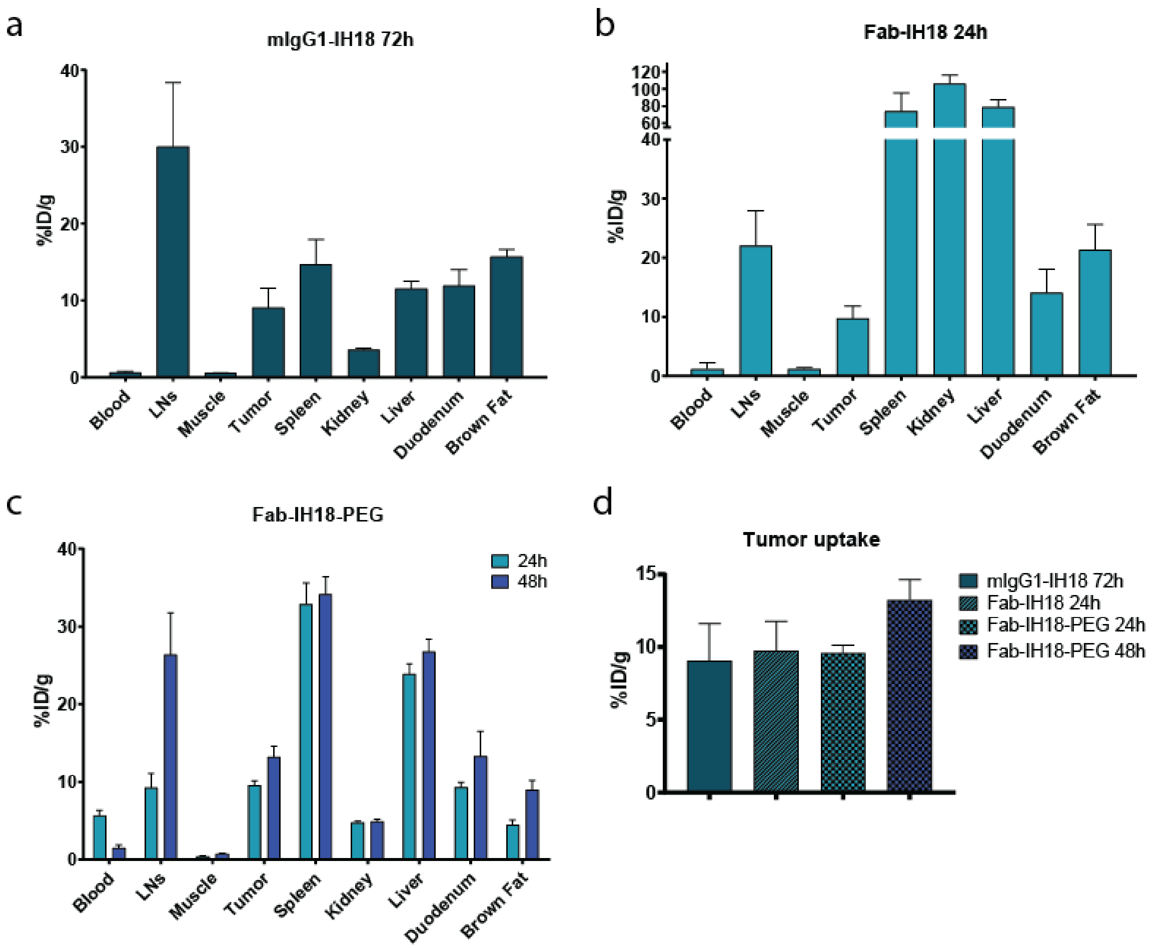

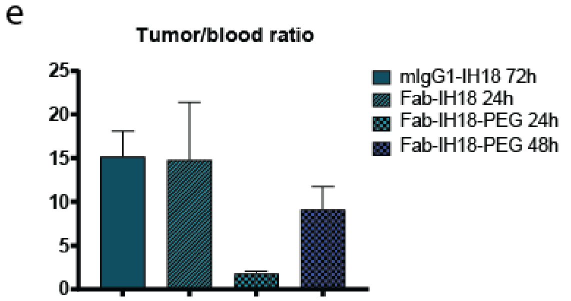

Figure S6. Biodistribution results of multi-modal PD-L1 imaging tools for SPECT. Biodistribution is shown **a)** at 72h for ^{111}In-labeled mIgG1, **b)** 24h for Fab and **c)** 24h and 48h for Fab-PEG$_{20kDa}$ PD-L1-IH18 for SPECT imaging. Tumor uptake values **(d)** and tumor/blood ratios **(e)** of the three constructs are compared directly at each time point. Data are shown as mean ± SD, n = 5.

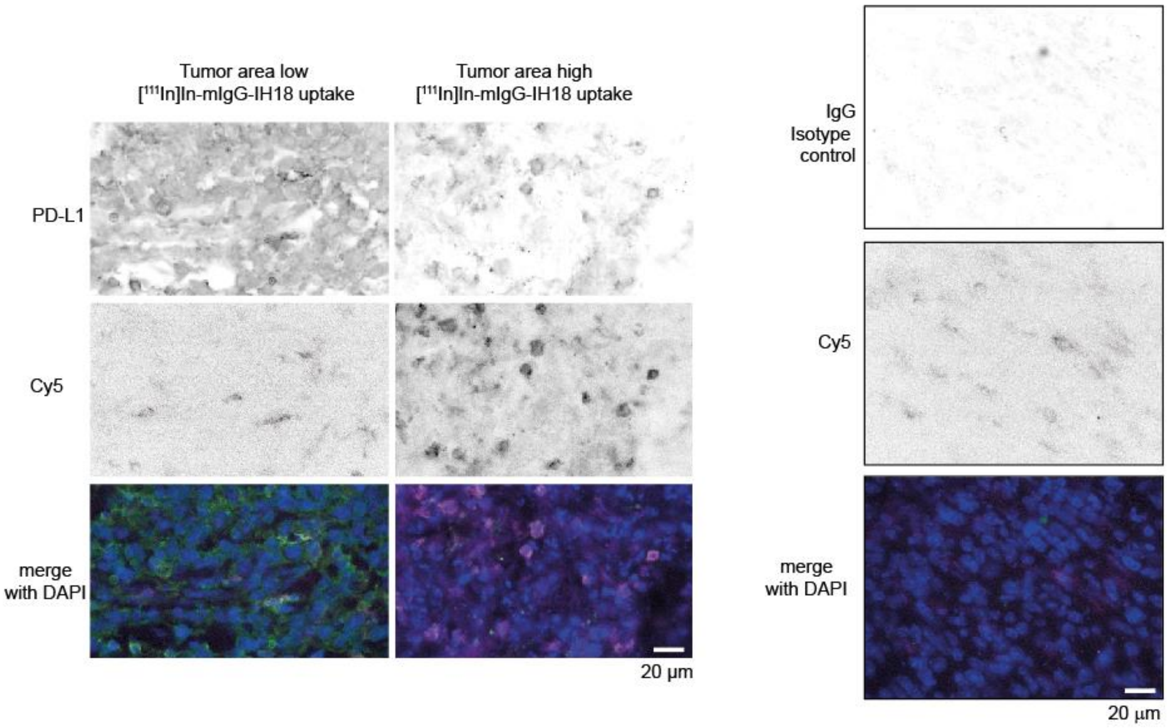

Figure S7. Fluorescence microscopy of tumor sections for mIgG1-IH18 localization. Fluorescence microscopy shows localization of mIgG1-IH18 with cellular resolution (DAPI in blue, Cy5 fluorescence in purple and PD-L1 immunofluorescent staining in green in the merge image). Isotype control for immunofluorescent staining shown on the right panels.

Table S1. Biodistribution results.

Antibodies (30 ug, 24h p.i.) were administered to BALB/c mice with 4T1 fatpad tumors (n = 5). Results are presented per tissue as mean ± SD (%ID/g).

Tissue	rIgG2a MIH5 (WT) DTPA (%ID/g)	mIgG1 MIH5 DTPA (%ID/g)	mIgG1 MIH5 IH20 (%ID/g)	mIgG1 MIH5 IH18 (%ID/g)
Blood	10.94 ± 2.86	2.91 ± 0.88	7.48 ± 0.95	9.36 ±1.38
LN Left	27.35 ± 2.80	18.54 ± 7.81	11.45 ± 6.30	21.03 ± 7.16
LN Right	18.92 ± 1.88	13.95 ± 1.68	9.96 ± 5.13	10.88 ± 12.35
Muscle	1.63 ± 0.30	1.16 ± 0.12	1.03 ± 0.07	1.25 ± 0.31
Tumor	16.52 ± 5.32	13.08 ± 1.02	15.78 ± 1.71	14.36 ± 2.37
Thymus	8.64 ± 0.55	6.94 ± 1.80	7.78 ± 1.16	15.74 ± 0.58
Lung	15.29 ± 1.24	8.65 ± 0.88	10.04 ± 0.69	12.91 ± 0.78
Spleen	13.96 ± 1.18	15.11 ± 2.67	12.11 ± 0.43	16.80 ± 0.68
Adrenal	10.03 ± 1.36	8.72 ± 0.87	7.44 ± 1.44	9.12 ± 0.69
Kidney	8.19 ± 1.21	5.44 ± 0.43	9.34 ± 0.38	8.19 ± 0.21
Liver	6.56 ± 0.51	20.65 ± 3.67	8.23 ± 0.43	9.14 ± 0.56
Duodenum	10.94 ± 0.52	9.04 ± 1.29	6.53 ± 1.69	7.42 ± 0.64
Colon	5.06 ± 0.10	3.88 ± 0.31	3.48 ± 0.24	5.25 ± 1.42
Brown Fat	22.69 ± 6.23	20.60 ± 3.99	14.05 ± 2.94	15.39 ± 2.60
Bone Marrow	5.32 ± 0.23	6.47 ± 1.52	5.69 ± 0.28	6.63 ± 1.17
Bone	1.74 ± 0.18	1.49 ± 0.14	1.46 ± 0.15	1.96 ± 0.47

Table S2. Results of PD-L1 biodistribution study.

Antibody-conjugates were administered to BALB/c mice with 4T1 fatpad tumors (n = 5). Biodistribution results are shown at their optimal time points for mIgG1-IH18 (72h), Fab-IH18 (24h) or Fab-IH-18-PEG (24h). Results are presented per tissue as mean ± SD (%ID/g).

Tissue	mIgG1-IH18 72h (%ID/g)	Fab-IH18 24h (%ID/g)	Fab-IH18-PEG 24h (%ID/g)
**Blood**	0.42 ± 0.17	0.31 ± 0.08	2.03 ± 0.30
**LN Left**	15.09 ± 3.61	7.51 ± 1.37	11.29 ± 1.60
**LN Right**	14.59 2.61	7.67 ± 1.15	13.61 ± 2.25
**Muscle**	0.59 ± 0.14	0.73 ± 0.21	0.87 ± 0.08
**Tumor**	7.95 ± 1.64	9.67 ± 0.84	16.55 ± 2.80
**Thymus**	7.60 ± 0.93	4.30 ± 0.16	3.59 ± 0.65
**Lung**	6.97 ± 3.33	2.66 ± 0.73	4.37 ± 0.62
**Spleen**	9.66 ± 0.68	10.11 ± 0.84	19.27 ± 1.55
**Adrenal**	5.50 ±1.56	4.50 ± 0.76	8.66 ± 1.79
**Kidney**	4.19 ± 0.70	78.36 ± 10.31	6.22 ± 0.75
**Liver**	8.12 ± 0.84	16.47 ± 1.19	18.09 ± 1.51
**Duodenum**	9.06 ± 2.73	4.89 ± 1.46	8.53 ± 1.12
**Colon**	2.54 ± 0.18	2.57 ± 0.24	3.52 ± 0.35
**Brown Fat**	17.18 ± 3.40	9.97 ± 2.32	9.58 ± 2.27
**Bone Marrow**	4.79 ± 0.98	5.10 ± 0.77	6.29 ± 1.06
**Bone**	1.02 ± 0.15	1.22 ± 0.14	1.36 ± 0.27
**Tumor/blood**	20.67 ± 8.60	32.68 ± 7.95	8.22 ± 1.32

Table S3. Biodistribution results of PD-L1 imaging study.
Antibody-conjugates were administered to BALB/c mice with 4T1 fatpad tumors (n = 5). Results are presented per tissue as mean ± SD (%ID/g).

Tissue	mIgG1-IH18 72h (%ID/g)	Fab-IH18 24h (%ID/g)	Fab-IH18-PEG 24h (%ID/g)	Fab-IH18-PEG 48h (%ID/g)
**Blood**	0.61 ± 0.17	1.13 ± 1.07	5.63 ± 0.74	1.53 ± 0.35
**LN (2x)**	30.00 ± 8.38	22.02 ± 5.90	9.25 ± 1.85	26.4 ± 5.38
**Muscle**	0.55 ± 0.03	1.15 ± 0.22	0.4 ± 0.08	0.76 ± 0.09
**Tumor**	9.04 ± 2.56	9.74 ± 2.01	9.58 ± 0.54	13.2 ± 1.41
**Thymus**	9.33 ± 0.66	11.76 ± 2.23	12.41 ± 0.23	3.1 ± 0.50
**Lung**	3.83 ± 0.85	9.41 ± 6.26	3.96 ± 0.68	3.29 ± 0.70
**Spleen**	14.69 ± 3.27	73.89 ± 21.59	32.87 ± 2.74	34.17 ± 2.25
**Kidney**	3.56 ± 0.19	106.54 ± 9.83	4.77 ± 0.16	4.89 ± 0.30
**Liver**	11.53 ± 1.00	78.74 ± 8.87	23.91 ± 1.27	26.79 ± 1.54
**Duodenum**	11.89 ± 2.09	14.05 ± 3.97	9.3 ± 0.64	13.26 ± 3.26
**Colon**	2.69 ± 0.27	5.22 ± 1.17	2.18 ± 0.12	3.51 ± 0.43
**Brown Fat**	15.70 ± 0.94	21.32 ± 4.27	4.5 ± 0.58	9.00 ± 1.18
**Bone Marrow**	3.72 ± 1.65	9.52 ± 1.31	4.94 ± 0.24	6.93 ± 0.75
**Bone**	1.09 ± 0.22	1.96 ± 0.38	1.1 ± 0.14	1.73 ± 0.13
**Tumor/blood**	15.16 ± 2.95	14.79 ± 6.60	1.75 ± 0.32	9.13 ± 2.61
